# Effects of muscle strength training with blood flow restriction on glomerular function in people with stage 3 chronic kidney disease

**DOI:** 10.1590/2175-8239-JBN-2026-0061en

**Published:** 2026-10-16

**Authors:** Antonio Filipe Pereira Caetano, Janyeliton Alencar de Oliveira, Amaro Wellington da Silva, Cyro Rego Cabral, Michelle Jacintha Oliveira Cavalcante, Maria do Socorro Cirilo-Sousa

**Affiliations:** 1 Universidade Federal de Alagoas, Instituto de Educação Física e Esporte, Maceió, AL, Brazil.; 2 Universidade Federal da Paraíba, João Pessoa, PB, Brazil.; 3 Universidade Federal de Alagoas, Hospital Universitário Professor Alberto Antunes, Maceió, AL, Brazil.; 4 Universidade Federal de Alagoas, Maceió, AL, Brazil.; 5 Universidade Federal de Alagoas, Faculdade de Medicina, Maceió, AL, Brazil.; 6 Universidade Regional do Cariri, Curso de Educação Física, Crato, CE, Brazil.

**Keywords:** Resistance Training, Vascular Occlusion, Renal Insufficiency, Biomarkers

## Abstract

**Introduction:**

Stage 3 chronic kidney disease (CKD) represents a critical juncture in clinical management. However, muscle strength training (MST) combined with blood flow restriction (BFR) has shown benefits as an adjunctive treatment, but its renal effects remain unclear.

**Objective:**

To analyze the effects of MST+BFR on markers of glomerular function in individuals with CKD-3.

**Method:**

A randomized, longitudinal clinical trial with 40 participants divided into 4 groups: control (G1); low-load MST (G2); high-load MST (G3); and low-load MST combined with BFR (G4). Serum creatinine, cystatin C, microalbuminuria, and estimated eGFR were used as dependent variables. The MST protocol was conducted over 12 weeks, 3 times per week, for 30–40 minutes, with 3 exercises (single- and multi-joint) per day at progressive intensities. Data analysis (intention-to-treat) was performed using the Shapiro-Wilk test (normality), paired *t*-test/Wilcoxon test (within-group comparisons), ANOVA/Kruskal-Wallis tests (between-group comparisons), Bonferroni test (*post hoc* corrections), and Cohen’s *d* (effect size) with a *p*-value ≤ 0.05.

**Results:**

The participants were predominantly male (52.5%) with low levels of physical activity (60%), hypertension (87.5%), and some with diabetes (37.5%). Group 4 showed a significant increase in albuminuria levels (*p* = 0.048), as well as significant differences compared with Group 1 (creatinine, *p* = 0.032) and Group 2 (creatinine, *p* = 0.036; and eGFR-Cr, *p* = 0.032).

**Conclusion:**

MST+BFR was not associated with severe adverse effects on serum creatinine/cystatin C levels, eGFR estimates, or urinary creatinine and microalbuminuria levels, but it may significantly increase albuminuria levels.

## INTRODUCTION

The kidneys perform numerous physiological functions, including hemodynamic, hormonal, hematological, skeletal, biochemical, electrolyte, and metabolic regulatory processes^1^. Consequently, when a prolonged, irreversible, and persistent impairment of renal function occurs, chronic kidney disease (CKD) develops^2^.

Disease progression can be assessed through the evaluation of glomerular function, which is determined by the excretion of metabolites (creatinine) and proteins (albumin) and/or blood cells during the clearance process. During filtration, albumin is normally retained while metabolic waste products resulting from protein metabolism are eliminated. Renal impairment is characterized by a reduction in nephron function^3^.

Renal injury promotes irreversible nephron loss and alterations in intrarenal renin–angiotensin system activity, contributing to increased blood pressure and glomerular blood flow. Simultaneously, adaptive hyperfiltration leads to proteinuria and renal fibrosis, ultimately resulting in diffuse segmental glomerulosclerosis. Therefore, the clearance of exogenous substances such as inulin, iothalamate-I, EDTA, Tc-DTPA, or iohexol allows a more accurate determination of glomerular filtration rate (GFR)^4^.

GFR can be defined as the volume of fluid filtered by the glomeruli per minute and is commonly estimated using predictive equations^5^. Stages 1–4 CKD (GFR >15 mL/min/ 1.73 m^2^) are generally managed through conservative or pre-dialysis treatment strategies, including pharmacological management and lifestyle modification. In contrast, stage 5 CKD (GFR <15 mL/min/1.73 m^2^) may require initiation of kidney replacement therapy, including hemodialysis, peritoneal dialysis, or kidney transplantation^6^.

Physical exercise has been recommended for individuals with CKD because of its potential to improve renal function^7^, promote favorable morphological adaptations^8^, enhance functional capacity^9^, increase lean body mass^10^, and improve perceived quality of life^11^. Nevertheless, sedentary behavior and insufficient physical activity levels remain highly prevalent among individuals with CKD^12^. Although the reasons for this phenomenon are not yet fully understood, they appear to be similar to those observed in the general adult population without chronic comorbidities^13^.

Among exercise modalities, resistance training (RT) combined with blood flow restriction (BFR) has gained attention because it can produce substantial gains in muscle mass and strength while requiring fewer exercises, shorter training durations, and lower exercise loads^14^. In CKD patients, previous studies have reported metabolic and blood pressure improvements, increased dynamic strength, enhanced functional capacity, benefits for arteriovenous fistula development, improved autonomic regulation, attenuation of GFR decline, favorable changes in endothelial function, vascular conductance, and venous compliance, as well as changes in inflammatory biomarkers, including IL-18, IL-10, IL-5, TNF-α, vasopressin, F2-isoprostane, NO_2_, and angiotensin-(1–7)^15^.

Despite these findings, significant gaps remain regarding the effects of exercise and its relationship with CKD, particularly among individuals with stage 3 CKD (CKD-3), characterized by a GFR ranging from 15 to 60 mL/min/1.73 m^2^. This stage represents a critical period in disease management because CKD is already firmly established, yet progression may still be attenuated through lifestyle modifications and behavioral interventions^16^. Previous investigations have primarily focused on either earlier stages (CKD-2) or more advanced stages (CKD-4 and CKD-5). In the former, CKD may remain underdiagnosed, whereas in the latter, severe vascular alterations may limit the safe implementation of BFR because of concerns regarding vascular injury and future arteriovenous fistula creation for hemodialysis treatment.

Furthermore, no studies have directly compared BFR-assisted resistance training with different loading strategies (high load, low load, and no load) to identify the relative benefits and potential risks associated with each resistance training modality. Existing evidence consistently demonstrates that resistance training performed at different intensities influences muscle strength, muscle hypertrophy, and biochemical parameters in populations without chronic diseases^17,18,19^. However, in individuals with CKD, clinical conditions such as dietary protein restrictions and pharmacological treatment may directly influence these adaptations. Therefore, evaluating the impact of a resistance training model employing different intensities may help identify effective exercise protocols specifically tailored to this population, particularly considering the preventive role of exercise in slowing CKD progression and preserving renal function.

Accordingly, the aim of this study was to investigate the effects of resistance training combined with BFR on glomerular function in individuals with stage 3 CKD.

## METHODS

This study was a randomized clinical trial with a longitudinal design, parallel groups, and repeated measures. The study protocol was approved by the Research Ethics Committee under approval number 7.159.681/2024 and was registered with the Brazilian Registry of Clinical Trials (ReBEC) under registration number RBR-7bt6dxb.

### Participants and sample

The sample consisted of adults and older adults of both sexes, aged between 35 and 75 years, diagnosed with CKD and presenting an estimated glomerular filtration rate (eGFR) between 30 and 59 mL/min/1.73 m^2^. Participants were recruited from the Urinary System Unit of a tertiary-care university hospital in Maceió, Alagoas, Brazil.

Sample size estimation was performed using G*Power software, assuming a 95% confidence interval, a 5% margin of error, and an estimated prevalence of CKD patients with eGFR between 30 and 59 mL/min/1.73 m^2^ receiving care at the institution between January and July 2024.

Considering a finite population of 197 individuals, a statistical power of 0.80, a significance level of 5%, an effect size of 0.25, four study groups, two repeated measurements, and a correlation coefficient of 0.50 between repeated measures, the minimum required sample size was estimated at 24 participants. To account for potential attrition, the final sample size was increased to 40 participants.

Randomization was performed using stratified block randomization through the Randomizer.org platform. Participants were equally allocated (25% each) into four groups: control group (G1): no resistance training; low-load resistance training group (G2); high-load resistance training group (G3); low-load resistance training combined with BFR (G4).

Groups were matched according to sex, physical activity level, CKD stage 3 subclassification, quality of life, and functional capacity.

A total of 40 participants were randomized, with 10 individuals assigned to each group. At the end of the 12-week intervention period, four participants withdrew from the study (two from G2 and two from G3) due to a pre-existing injury (n = 1), anemia (n = 1), and transportation difficulties (n = 2). The flowchart of the study participants can be seen in Figure 1. Overall adherence to the training protocol was 90%.

**Figure 1:**
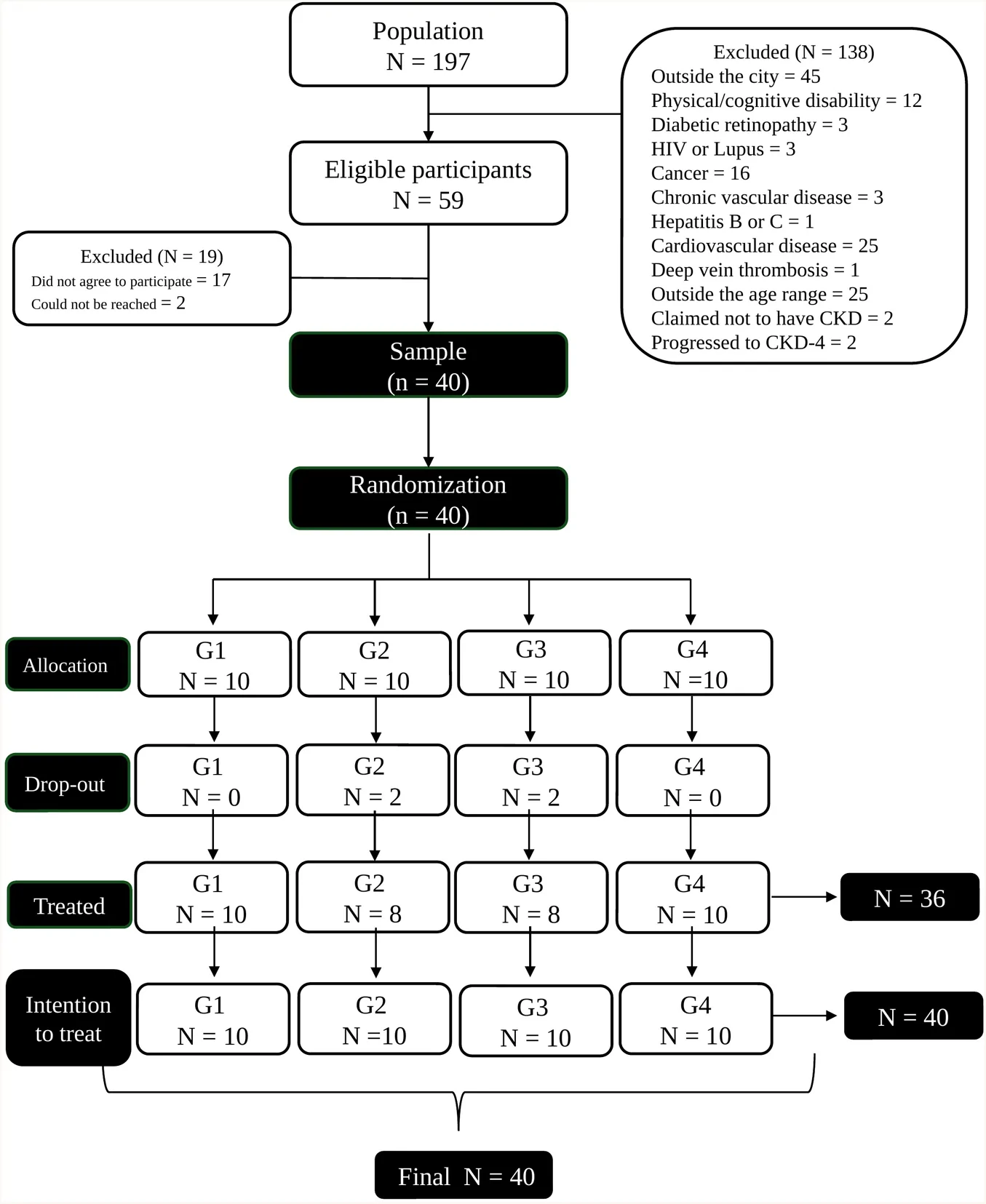
Flowchart of participants through selection, randomization, and intervention.

Abbreviations – G1: control group; G2: low-load group; G3: high-load group; G4: blood flow restriction group.

Sources – Authors, 2025; CONSORT, 2025.

The mean attendance rate among all participants was 86.5%, reaching 96.5% in G4, 81.7% in G2, and 81.6% in G3.

### Eligibility criteria

Eligible participants were individuals with CKD who lived near the study site, signed written informed consent, received medical clearance from their nephrologist, and did not have any of the following conditions: neurodegenerative or infectious diseases (including active HIV/AIDS infection and systemic lupus erythematosus); symptomatic heart failure classified as New York Heart Association (NYHA) class II, III, or IV; sepsis within the previous 12 months; active treatment for hepatitis B or C; resting blood pressure of ≥160/100 mmHg before exercise; coagulation disorders or signs of thrombophlebitis; surgery within the previous three months; severe arrhythmia, angina, cerebrovascular disease, or cardiovascular disease; pulmonary congestion or peripheral edema; previous upper-extremity surgery or vascular access procedures; an ankle-brachial index (ABI) of <0.91 or >1.30.

Individuals who failed to attend evaluations or questionnaire assessments or who did not obtain medical authorization were excluded from the study.

### Questionnaires

Participants completed a sociodemographic and lifestyle questionnaire including information regarding age, sex, educational level, self-reported race/ethnicity, marital status, tobacco use, alcohol consumption, use of other substances, and associated comorbidities.

Physical activity level was assessed using the International Physical Activity Questionnaire (IPAQ)^20^. Participants reporting less than 150 minutes of physical activity per week were classified as insufficiently active, whereas those reporting at least 150 minutes per week were classified as physically active, according to the Brazilian Physical Activity Guidelines^21^.

### Glomerular function markers

Blood and urine samples were collected for the assessment of glomerular function. Serum creatinine, cystatin C, and urinary microalbuminuria were analyzed using kinetic colorimetric, nephelometric, and turbidimetric methods, respectively. No fasting period was required before sample collection.

Three blood collection tubes and one urine specimen container were obtained from each participant. Two blood samples were collected without anticoagulant, and one sample was collected using EDTA/heparin for additional analyses.

Estimated GFR based on serum creatinine (eGFR-Creatinine) was calculated using the CKD-EPI equation: 142 × min (SCr/κ,1)^α × max (SCr/κ,1)^−1.200 × 0.9938^ Age × 1.012 (if female)^22^, where SCr is the serum creatinine concentration.

Estimated GFR based on cystatin C (eGFR-Cystatin C) was calculated using the equation: 133 × min (CysC/0.8,1)^ −0.499 × max (CysC/0.8,1)^−1.328 × 0.996^Age × 0.932 (if female)^23^, where CysC represents the serum cystatin C concentration.

### Blood flow restriction (BFR) assessments

Resting systolic and diastolic blood pressure was measured after 10 minutes of seated rest using a Premium ML177 sphygmomanometer. Participants presenting blood pressure values of ≥160/105 mmHg were not allowed to perform the intervention session.

Heart rate and peripheral oxygen saturation were assessed after 10 minutes of rest using a G-Tech 300C pulse oximeter. Cutoff values for exercise participation were >150 beats/min and <70% oxygen saturation.

The ABI was measured as a preventive screening procedure, with acceptable values ranging from 0.91 to 1.30.

To determine the appropriate levels of BFR, the auscultatory pulse of the brachial (upper limbs) and dorsalis pedis (lower limbs) arteries was assessed using a Premium pneumatic tourniquet (model ML177), secured in the proximal axillary region, with a width of 52 × 14.5 cm, and in the proximal thigh region, with a width of 78 × 21 cm, with a reading of 300 mmHg. Auscultation of the insonation pressure was performed using a MEDPEJ portable vascular Doppler device, in accordance with the recommendations of Gil et al.^24^.

### Ten-repetition maximum test

The 10-repetition maximum (10RM) test was conducted according to the American College of Sports Medicine guidelines^25^ and adapted guidelines for clinical populations^26^. Assessments were performed during week 1, week 6, and after 12 weeks of training.

Participants first performed a warm-up without external load to familiarize themselves with the movement pattern and prepare the musculoskeletal system. Subsequently, a preliminary load was selected, and participants performed sets of 10 repetitions with progressive increases in load resistance until concentric failure was reached.

The highest successfully completed load was recorded as the participant’s 10RM.

To minimize measurement error, the following procedures were adopted: all assessments were conducted at the same time of day; standardized instructions were provided before testing; participants received technical guidance regarding exercise execution; and standardized verbal encouragement was provided throughout testing.

### Intervention protocol

The training protocol used is described in another study^27^. The intervention took place over 12 weeks (36 sessions), with 3 sessions per week, for a total of 36 sessions. Each session included stretching (unilateral elbow flexion behind the back; unilateral knee flexion; hip flexion with fingers touching the floor; hip flexion on the stall bars; calf stretch against the wall), warm-up on a cycle ergometer (10 min, without intensity control), and 9 strength exercises, namely: đ straight-arm dumbbell bench press, hip extension, triceps pushdown, seated row, leg curl, barbell curl, 45° leg press, dumbbell shoulder press, and calf raises on the 45° leg press.

The protocol was periodized into 4 cycles, with loads, sets, and repetitions adjusted to ensure a similar training effect across all groups. Thus, for G2 and G3, in the first cycle (weeks 1–3), 3 sets of 10–12 repetitions were performed, with 1 minute of rest, using loads of 30% and 60% of the 10RM, respectively. In the second cycle (weeks 4–6), the same number of sets, repetitions, and rest interval was maintained, but with loads adjusted to 35% and 70% of the 10RM. In the third cycle (weeks 7–9), 3 sets of 8–10 repetitions were performed, with loads of 40% and 80% of the 10RM. Finally, in the fourth cycle (weeks 10–12), 3 sets of 10–12 repetitions were performed, with 1 minute of rest and loads of 40% and 80% of the 10RM.

For the G4 group, in all cycles, 4 sets of each exercise were performed, with 15 repetitions and a load of 30% of the 10RM. In the 7th week, the BFR value was adjusted following a reassessment of the insonation pressure. The exercises followed the timing set by a DIGIPOM version 1.2.0 digital metronome, emitting a digital sound in the Indigo theme, with a rhythm of 2 beats per measure (2/4 time), equivalent to 48 beats per minute. Blood vessel restriction was applied in a conditioning manner (during training), at 50% of the diastolic blood pressure, and performed continuously. For BFR, the Hyper Recovery device (nylon material) was used, with dimensions of 68 x 8 cm (upper limbs) and 108 × 10 cm (lower limbs), a mercury manometer with a range of 0–300 mmHg, or an adapted premium device (nylon material), with dimensions of 52 × 6 cm (upper limbs) or 76 × 17 cm (lower limbs), and a mercury manometer with a range of 0–300 mmHg, depending on the type of exercise performed.

Blood pressure (BP) and heart rate (HR) were measured before and after each session throughout the study intervention period. During the intervention sessions, exercise intensity was monitored using the Borg Rating of Perceived Exertion Scale (RPE) after completing the prescribed number of repetitions for each exercise, with an increase of 0.5 to 1 kg when a rating below 7 on the scale was reported.

Finally, the affective experience scale for physical exercise was administered before and after the training, as proposed by Hardy and Rejeski^28^.

### Statistical analysis

An intention-to-treat approach was adopted. All randomized participants completed baseline and post-intervention assessments; therefore, no data imputation procedures were required.

Statistical analyses were performed using the R software (R Core Team, 2024).

Data normality was assessed using the Shapiro–Wilk test. For normally distributed variables, within-group comparisons were performed using paired *t* tests. Non-normally distributed variables were analyzed using the Wilcoxon signed-rank test.

Between-group comparisons at post-intervention were conducted using one-way analysis of variance (ANOVA) for normally distributed variables, followed by Tukey’s *post hoc* test. For non-normally distributed variables, the Kruskal–Wallis test was used, followed by Dunn’s multiple comparison test with Bonferroni correction.

Linear mixed-effects models with random intercepts for participants were fitted to account for repeated measurements over time. Fixed-effect significance was estimated using the Satterthwaite approximation implemented in the lmerTest package.

Effect sizes were calculated using Cohen’s *d*. For within-group analyses, paired-sample effect sizes and 95% confidence intervals were computed. For post-intervention between-group comparisons, Cohen’s *d* for independent samples was calculated using G4 as the reference group. Effect sizes were interpreted according to Rhea’s classification: *d* < 0.35, trivial; 0.35 ≤ *d* < 0.80, small; 0.80 ≤ *d* < 1.50, moderate; *d* ≥ 1.50, large.

Statistical significance was established at *p* ≤ 0.05.

### Clinical monitoring

Participants remained under nephrological supervision throughout the 12-week intervention period, with monthly clinical consultations.

Additionally, every four weeks, participants were interviewed regarding discomfort, adverse symptoms, musculoskeletal injuries, and barriers to protocol adherence.

Any suspected vascular, thrombotic, cardiovascular, or renal complication would result in immediate referral to the hospital’s nephrology service for further evaluation.

Participants experiencing adverse events that contraindicated continued participation were withdrawn from the study, and all events were documented and reported to the Research Ethics Committee in accordance with Brazilian National Health Council Resolutions 466/2012 and 510/2016.

## RESULTS

A total of 40 individuals participated in the study (52.5% men; mean age: 58 ± 8.9 years). Sociodemographic and health-related characteristics are presented in Table 1. Most participants had hypertension (87.5%), 37.5% had diabetes mellitus, and 60% were classified as having low levels of physical activity.

**Table 1:** Sociodemographic and lifestyle characteristics of people with stage 3 CKD.

Variables	G1 N = 10 (%)	G2 N = 10 (%)	G3 N = 10 (%)	G4 N = 10 (%)	Overall N = 40 (%)
Sex					
Men	5 (50.0)	5 (50.0)	6 (60.0)	5 (50.0)	21 (52.5)
Women	5 (50.0)	5 (50.0)	4 (40.0)	5 (50.0)	19 (47.5)
Skin color					
Brown	5 (50.0)	5 (50.0)	1 (10.0)	7 (70.0)	18 (45.0)
Black	3 (30.0)	2 (20.0)	4 (40.0)	1 (10.0)	10 (25.0)
White	2 (20.0)	2 (20.0)	4 (40.0)	1 (10.0)	9 (22.5)
Indigenous	–	1 (10.0)	1 (40.0)	1 (10.0)	3 (7.5)
Marital status					
Single	5 (50.0)	2 (20.0)	2 (20.0)	2 (20.0)	11 (27.5)
Married	4 (40.0)	7 (70.0)	7 (70.0)	6 (60.0)	24 (60.0)
Widow	1 (10.0)	–	–	1 (10.0)	2 (6.25)
Divorced	–	1 (10.0)	1 (10.0)	1 (10.0)	3 (6.25)
Employed					
Yes	3 (30.0)	2 (20.0)	3 (30.0)	6 (60.0)	14 (35.0)
Level of education					
Incomplete elementary school	3 (30.0)	7 (70.0)	5 (50.0)	2 (20.0)	17 (42.5)
Complete elementary school	2 (20.0)	1 (10.0)	–	3 (20.0)	6 (15.0)
High school	2 (20.0)	–	5 (50.0)	3 (30.0)	10 (25.0)
Illiterate	1 (10.0)	1 (10.0)	–	–	2 (15.0)
Higher education	2 (20.0)	1 (10.0)	–	2 (20.0)	5 (2.5)
Diabetes					
Yes	4 (40.0)	4 (40.0)	5 (50.0)	2 (20.0)	15 (37.5)
Hypertension					
Yes	8 (80.0)	8 (80.0)	9 (90.0)	10 (100.0)	35 (87.5)
Other diseases					
Yes	7 (80.0)	4 (40.0)	3 (30.0)	4 (40.0)	18 (45.0)
Smoking					
Yes	1 (10.0)	4 (40.0)	3 (30.0)	2 (20.0)	10 (25.0)
No	9 (90.0)	5 (50.0)	6 (60.0)	8 (80.0)	28 (70.0)
Former smoker	–	1 (10.0)	1 (10.0)	–	2 (5.0)
Alcohol					
Yes	–	3 (30.0)	3 (30.0)	3 (30.0)	9 (22.5)
No	9 (90.0)	4 (40.0)	5 (50.0)	3 (30.0)	21 (52.5)
Former drinker	1 (10.0)	3 (30.0)	2 (20.0)	4 (40.0)	10 (25.0)
CKD Stage					
3a	4 (40.0)	3 (30.0)	5 (50.0)	1 (10.0)	13 (32.5)
3b	6 (60.0)	7 (70.0)	5 (50.0)	9 (90.0)	27 (67.5)
Physical Activity					
Sedentary	5 (50.0)	5 (50.0)	1 (10.0)	2 (20.0)	13 (32.5)
Insufficiently active	2 (10.0)	3 (30.0)	4 (40.0)	2 (20.0)	11 (27.5)
Active	–	–	3 (30.0)	3 (20.0)	6 (15.0)
Very active	3 (40.0)	2 (20.0)	2 (20.0)	3 (30.0)	10 (25.0)

Abbreviations – G1: control group; G2: low-load group; G3: high-load group; G4: blood flow restriction group; N: number; %: percentage; CKD: chronic kidney disease.

Source – Authors, 2025.

Within-group comparisons following the intervention are presented in Table 2, and a summary of these findings is shown in Figure 2.

**Table 2:** Comparison of intragroup means/medians for markers of glomerular function 12 weeks into the intervention protocol.

Variables	G1					G2					G3					G4				
	T0	T1	∆	1-β	*p*- value	T0	T1	∆	1-β	*p*- value	T0	T1	∆	1-β	*p*- value	T0	T1	∆	1-β	*p*- value
Cr (mg/dL)	1.34 ± 0.26	1.39 ± 0.26	+0.05	0.14	0.010^*^	1.30 ± 0.41	1.29 ± 0.26	−0.01	0.05	0.322	1.57 ± 0.52	1.71 ± 0.64	+0.14	0.08	0.031^*^	1.90 ± 0.7	2.06 ± 0.92	+0.16	0.11	0.066
Cist C (mg/L)	1.63 ± 0.43	1.51 ± 0.34	−0.12	0.10	0.071	1.57 ± 0.46	1.47 ± 0.40	−0.10	0.05	0.556	1.65 ± 0.57	1.67 ± 0.47	+0.02	0.06	0.845	1.8^‡^ [1.5–1.9]	1.9 ± 0.6	+0.10	0.05	0.049
MAU_Cr (mg/dL)	69.0 ± 30.4	106.1 ± 40.5	+37.1	0.31	0.005^*^	73.0^‡^ [44.0–181.0]	89.0 ± 41.3	+16.0	0.10	0.463	107.4 ± 78.3	111.3 ± 51.9	+3.9	0.05	0.808	115.7 ± 45.9	92.4 ± 42.8	−23.3	0.07	0.190
MAU_Alb (mg/L)	127.8 ± 84.9	144.5^‡^ [9.9–593.8]	+16.7	0.07	0.492	34.6^‡^ [5.9–1238.0]	178.7^‡^ [7.0–1329]	+144.1	0.12	0.598	40.7^‡^ [6.5–1866]	24.7^‡^ [7.0–1365]	−16.0	0.09	0.315	101.9^‡^ [9.0–709.7]	174.0 ± 171.0	+72.1	0.06	0.048^*^
MAU_RAC (mg/g creatinine)	218.5^‡^ [16.0–609.0]	131.5 ± 103.8	−87.0	0.10	0.048^*^	44.0^‡^ [8.0–2211]	42.5^‡^ [8.0–1642]	−1.5	0.11	0.050^*^	56.0^‡^ [7.0–1048]	23.0^‡^ [6.0–1219]	−33.0	0.11	1.000	103.8 ± 53.7	127.0^‡^ [10–173]	+22.2	0.05	0.845
TFG-Cr (mL/min/ 1.73 m^2^)	46.4 ± 9.7	44.5 ± 9.8	−1.9	0.78	0.010^*^	57.0^‡^ [51.0–60.0]	52.1 ± 6.2	−4.9	0.05	0.495	45.7 ± 13.6	43.4 ± 15.4	−2.3	0.40	0.120	39.6 ± 13.2	37.4 ± 14.9	−2.2	0.55	0.066
TFG-C (mL/min/ 1.73 m^2^)	47.4 ± 19.3	52.9 ± 16.1	+5.5	0.06	0.020^*^	48.1 ± 14.9	51.8^‡^ [48.6–60.9]	+3.7	0.08	0.089	49.7^‡^ [27.6–59.6]	41.0 ± 15.4	−8.7	0.25	0.179	38.1 ± 16.7	33.6^‡^ [22.8–38.3]	−4.5	0.05	0.781

Abbreviations – G1: control group; G2: low-intensity group; G3: high-intensity group; G4: blood flow restriction group; T0: pre-intervention; T1: post-intervention; Cr: serum creatinine; Cyst C: cystatin C; MAU_Cr: microalbuminuria–creatinine; MAU_Alb: microalbuminuria–albumin; MAU_RAC: microalbuminuria–albumin/creatinine ratio; eGFR-Cr: estimated glomerular filtration rate (creatinine); eGFR-C: estimated glomerular filtration rate (cystatin C).

Note – (‡): median [interquartile range];

(*): *p*-value < 0.05 within groups; 1-β: *post hoc* effect; ∆: difference between time points.

Source – Authors, 2025.

**Figure 2:**
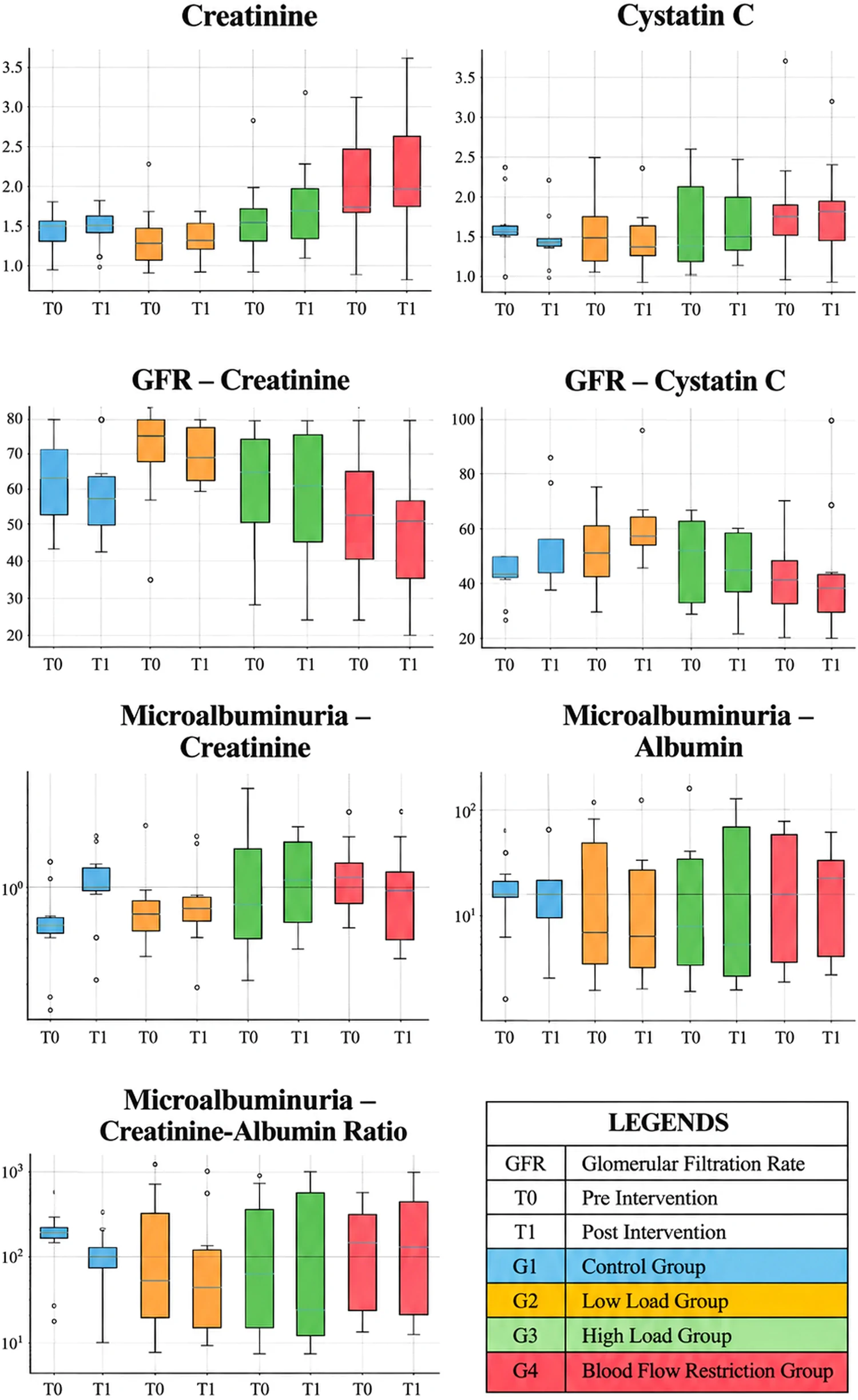
Boxplot of glomerular function results within groups and between time points.

In the control group (G1), statistically significant changes were observed in serum creatinine (*p* = 0.010; *d* = 1.13), urinary microalbuminuria-to-creatinine ratio (*p* = 0.005), urinary creatinine-to-albumin ratio (*p* = 0.048; *d* = 1.15), creatinine-based estimated glomerular filtration rate (eGFR-Cr) (*p* = 0.048; *d* = −0.84), and cystatin C-based estimated glomerular filtration rate (eGFR-CysC) (*p* = 0.020; *d* = 0.89). In all cases, effect sizes were considered large. However, only the changes in the urinary creatinine-to-albumin ratio and eGFR-CysC represented clinically favorable outcomes.

In the low-load resistance training group (G2), improvement was observed only in the urinary microalbuminuria-to-creatinine ratio, showing a trend toward statistical significance (*p* = 0.050; *d* = 0.80), with a large effect size.

In the high-load resistance training group (G3), a significant increase in serum creatinine concentration was observed (*p* = 0.031; *d* = 0.80), also corresponding to a large effect size.

In the BFR group (G4), urinary albumin levels increased significantly (*p* = 0.048; *d* = −0.40), with a moderate effect size.

Between-group comparisons were performed using G4 as the reference group (Table 3). Significant differences were observed for serum creatinine when G4 was compared with G1 (*p* = 0.032; *d* = 0.62) and G2 (*p* = 0.036; *d* = 0.77), both representing moderate effect sizes.

**Table 3:** Comparison of glomerular function between the groups 12 weeks into the intervention protocol.

Variables	G1 × G4				G2 × G4				G3 × G4			
	Coef	*p*-value	1-β	95%CI	Coef	*p*-value	1-β	95%CI	Coef	*p*-value	1-β	95%CI
Cr (mg/dL)	−0.479	0.032*	0.05	−0.917; −0.042	−0.479	0.036*	0.20	−0.928; −0.031	−0.170	0.457	0.05	−0.617; 0.277
Cist C (mg/L)	−0.277	0.216	0.13	0.715; 0.162	−0.265	0.250	0.06	−0.716; 0.186	−0.121	0.597	0.05	−0.570; 0.328
MAU_Cr (mg/dL)	−8.254	0.660	0.07	−45.066; 28.558	−13.818	0.474	0.05	−51.655; 24.018	7.531	0.695	0.14	30.150; 45.212
MAU_Alb (mg/L)	−32.053	0.829	0.23	−323.194; 259.088	130.696	0.392	0.05	−168.418; 429.810	189.818	0.212	0.24	312.202; 1509.892
MAU_RAC (mg/g creatinine)	−98.374	0.563	0.05	−431.312; 234.565	140.96	0.419	0.07	−201.100; 483.023	134.83	0.438	0.23	−205.84; 475.512
TFG-Cr (mL/min/1.73 m^2^)	6.213	0.244	0.28	−4.28; 16.674	11.284	0.039*	0.96	0.547; 22.022	3.425	0.530	0.16	−5.04; 16.17
TFG-C (mL/min/1.73 m^2^)	10.482	0.190	0.11	−5.195; 26.160	11.824	0.150	0.40	4.28; 27.933	2.892	0.724	0.05	−13.151; 18.936

Abbreviations – G1: control group; G2: low-intensity group; G3: high-intensity group; G4: blood flow restriction group; T0: pre-intervention; T1: post-intervention; Cr: serum creatinine; Cyst C: cystatin C; MAU-Cr: microalbuminuria–creatinine; MAU_Alb: microalbuminuria–albumin; MAU_RAC: microalbuminuria–albumin/creatinine ratio; eGFR-Cr: estimated glomerular filtration rate (creatinine); eGFR-C: estimated glomerular filtration rate (cystatin C).

Note – (*): *p*-value < 0.05 between groups; 1-β: *post hoc* effect; ∆: difference between time points.

Source – Authors, 2025.

For creatinine-based estimated glomerular filtration rate (eGFR-Cr), a significant difference was observed between G2 and G4 (*p* = 0.039; *d* = 1.29), corresponding to a large effect size.

No statistically significant between-group differences were identified for serum cystatin C, urinary microalbuminuria, urinary albumin concentration, albumin-to-creatinine ratio, or cystatin C-based estimated glomerular filtration rate.

The overall attendance rate for the training sessions was 86.5%. The highest adherence was observed in the BFR group (G4), which achieved a mean attendance rate of 96.5%.

Regarding affective responses to exercise, only the high-load resistance training group (G3) demonstrated a significant improvement in affective perception scores (*p* = 0.031). Although mean values also improved in G2 and G4, these changes did not reach statistical significance.

Reported adverse events were mild and transient. Participants described occasional neck pain during the first weeks of training, burning sensations in the arms, limb fatigue, and skin itching. No severe adverse events, cardiovascular complications, thrombotic events, or clinically relevant renal complications were reported during the intervention period.

## DISCUSSION

After 12 weeks of RT, it was observed that participants who followed the BFR protocol did not show significant changes in serum creatinine and cystatin C levels, nor in GFR estimates (eGFR) based on these markers.

This may have occurred due to the need for rapid generation of muscular energy (ATP), such that creatine phosphate (phosphocreatine) is broken down into creatinine in the bloodstream. Thus, due to constant muscle activity during the tasks, creatinine excretion is continuous throughout the body^1,29^. However, when problems arise in the renal filtration process, the incidence of damage to the glomerular basement membrane becomes latent^2^.

RT tends to increase the use of ATP (adenosine triphosphate) molecules through oxidative phosphorylation in the mitochondria, anaerobic glycolysis, or substrate-level phosphorylation in the cytoplasm and mitochondria^30^. This system is acutely triggered by the central nervous system, which promotes muscle activation through the recruitment of type I motor units (slow-twitch, red) and, subsequently, type II fibers (fast-twitch, white)^31^.

However, when using the BFR method, this system is enhanced by hypoxia, which accelerates the recruitment of fast-twitch fibers, triggering a cascade effect that increases metabolic changes and stimulates the release of hormones such as testosterone, GH, and cortisol^32^. The physiological impacts described, associated with mechanical stresses that induce micro-shear injuries in muscle fibers, may facilitate gains in muscle hypertrophy and strength^33^. This may explain the non-statistically significant increase in serum creatinine levels in groups G3 and G4 in the present study, considering that factors such as exercise intensity, number of repetitions, and rest intervals directly influence energy metabolism. Furthermore, compared with G1 and G2, G4 showed a statistically significant difference in serum creatinine levels, although these two groups exhibited differing trends for this metabolite: an increase (G1) and a decrease (G2) in mean values following the intervention period.

This hypothesis is supported by the results for creatinine-based eGFR, where Group 1 was the only study arm to show differences (decreases in mean values) between time points, although it did not show a statistically significant difference compared with Group 4. In contrast, Group 2 showed a decrease in the mean values of the eGFR-creatinine estimate and a significant difference compared with Group 4. Thus, even though the equation accounts for sex and age, the results may indicate that RT+BFR can help mitigate the significant increase in serum creatinine and the decrease in eGFR-creatinine in people with CKD-3.

The use of the BFR method in people with CKD-3 has not yet been extensively evaluated in studies. For patients in stage 2, Corrêa et al.^34^ analyzed 229 patients using a 6-month MST protocol and found that creatinine levels remained stable with BFR but increased at other intensities. The same author, in another analysis involving 105 individuals with CKD-2, proposed an MST model with and without BFR and observed a reduction in eGFR and uremic levels, as well as an improvement in the inflammatory profile and the Klotho-FGF23 axis — a key marker for controlling phosphorus levels and mitigating cardiovascular and bone damage — in those exposed to exercise compared with the control group, which, in contrast, showed progression to CKD-3 by the end of the study^35^. These results, combined with our findings regarding the effects on eGFR, may indicate that MST is an important ally in slowing the accelerated progression of CKD in terms of glomerular filtration rate, with exercise intensity being a factor to consider, given that low-intensity training was shown to significantly reduce eGFR.

In this same perspective, Deus et al.^36^, while analyzing cardiac autonomic function in 105 individuals with CKD-2, also observed improvements in halting the decline in renal function, based on potential positive responses in redox balance, the inflammatory enzymes paraoxonase 1 (PON1) and myeloperoxidase (MPO), and in cardiovascular autonomic modulation, with the use of RT with or without BFR. Additionally, another study by Corrêa et al.^37^ involving 90 adult and elderly patients, changes in the expression levels of angiotensin-1, vasopressin, and nitric oxide were identified in the BFR groups, a situation that could affect elevations in creatinine levels given the body’s natural metabolic response. In line with this, these studies, which evaluated cardiorenal interactions in greater depth, may help explain the possible fluctuations in serum creatinine and eGFR levels in CKD-3, reinforcing the role of RT as a stressor and potential modulator of hemodynamic mechanisms, the sympathetic nervous system, endothelial dysfunction, NO_2_ expression, systemic inflammation, the renin-angiotensin system, and electrolyte imbalances, independent of the use of BFR.

Without the use of RT+BFR, previous studies have already reported improvements in eGFR, serum creatinine levels, and proteinuria in protocols that combined RT with aerobic training of varying intensities and the use of exercise equipment, while also analyzing different stages of CKD, from CKD-2 to CKD-4^38^. The failure to isolate CKD stage 3 likely prevents the identification of more precise responses regarding the effects of RT in this population, and the association with high-intensity aerobic training may mask potential results in terms of eGFR.

However, due to the influence exerted by muscle mass, protein intake, hydration status, and regular physical exercise practices on serum creatinine levels, cystatin C becomes an essential complementary biomarker for assessing glomerular function in CKD. Cystatin C is present in various cells of the body, is responsible for protein degradation processes, and functions as a regulator of protease activities; it contributes to the remodeling of the extracellular matrices, aids in regulating inflammatory disorders, contributes to immune protection, and acts in the degradation of intracellular proteins^39^.

Thus, under normal conditions, cystatin C is not significantly excreted during glomerular filtration, given its nearly complete reabsorption and degradation in the renal tubules^40^. In the context of exercise training, serum levels of this protein may increase during more intense activities, especially due to situational complications such as muscle injury, fluid loss, and elevated inflammatory markers, such as IL-6 and TNF-α. However, chronically, exercise training tends to minimize the increase in cystatin C expression, helping to stabilize inflammatory levels and leading to improved eGFR^41^.

In our findings, there was no significant difference in the mean values of cystatin C expression in any group after 12 weeks of exposure to the proposed protocol.

This is an interesting finding, given that, regardless of the RT load, muscle stress and the pressure exerted on blood vessels may not be sufficient to cause significant changes in this protein. However, it is worth mentioning the physiological characteristics of people with CKD, who tend to have high blood pressure, which, in some cases, can lead to impaired bone function and high fluid retention, especially in stage 3. This set of clinical conditions may influence the body’s slower adaptation to the adaptive responses promoted by MST in regulating serum cystatin C expression and glomerular filtration. Côrrea et al.^34^ also identified an increase in cystatin levels in their protocol for individuals with CKD-2, which may, in fact, indicate a tendency for this protein to increase during short-term training (3 to 6 months).

However, the lack of exposure to RT may have contributed to the positive and significant changes in the estimated eGFR-Cystatin in Group 1, where an increase in eGFR was observed; conversely, fluctuations in mean values were observed in the other study groups, though these were not statistically significant.

Given that eGFR is a marker for determining the progression of CKD stages, it is important to note that the only group that changed its eGFR classification stratum after the intervention period was Group 3, moving from stage 3a to stage 3b, regardless of whether creatinine or cystatin C was used for the calculation. Group 1 would change strata only (from 3a to 3b) when using eGFR-creatinine.

To detect this accelerated loss of renal function, the microalbuminuria test, which measures urinary excretion, is an important tool. The use of albumin as a marker provides an acute indication of glomerular damage, particularly due to associated conditions such as diabetes mellitus (DM) and hypertension (HTN), but it is also present in other comorbidities such as HIV, lupus, hepatitis, and glomerulonephritis. Because it is present in blood plasma, albumin helps maintain oncotic pressure balance, transports water-soluble substances (thyroxine, cortisol, free fatty acids, bilirubin, calcium, magnesium, and anti-inflammatory drugs), replenish amino acids during metabolic support, regulate blood pH levels, and minimize oxidative stress by neutralizing the action of free radicals^41^. In our findings, the urinary albumin-to-creatinine ratio showed significant improvements in G1 and G2; however, regardless of the group in which these microalbuminuria results were identified, the mean values were outside the normal range (< 30 mg/L).

The effect of RT on microalbuminuria is primarily related to the clinical conditions of the participants. Thus, higher-intensity training sessions yield more significant changes in microalbuminuria levels, affecting renal blood flow, increasing hemodynamic parameters, elevating oxidative stress, and altering inflammatory responses. However, when performed at more controlled intensities and on a chronic basis, exercise can help improve endothelial function and, consequently, regulate blood pressure and reduce microalbuminuria levels.

In this regard, we can consider the hypothesis that the use of the BFR method, due to vascular occlusion and the consequent accumulation of metabolites, may have exacerbated the negative and significant changes in albuminuria levels. Thus, vasoconstriction may have reduced the supply of oxygen and nutrients, increasing peripheral vascular resistance and BP values, which, in turn, by activating the sympathetic nervous system, contributed to hormonal changes (endothelin-1, angiotensin II, vasopressin, and catecholamines)^30,33^.

Hamada et al.^42^, who combined resistance training and aerobic exercise in individuals undergoing conservative treatment (CKD-2–CKD-4), observed a statistically significant decrease in proteinuria levels in these patients. However, these results were not replicated by Hiraki et al.^43^ and Zhang et al.^44^, who, although they observed an increase in proteinuria levels in the experimental group, did not find statistical significance. Regarding studies using the BFR method, this marker has not been included in existing analyses.

In the results of our investigation, no association was found between glomerular function and the participants’ glycemic conditions. However, Deus et al.^45^, when examining individuals with CKD-2 using an RT model with or without BFR over 6 months, observed that, while there was a decrease in eGFR, participants also showed improvements in glycemic homeostasis levels, hormonal mediators of glucose uptake, and their immunometabolic profile. These findings point to an association between metabolic and renal mechanisms in slowing the progression of CKD and reinforce RT as an essential modulator for additional gains in conservative treatment, particularly in the management of underlying conditions such as DM and HTN.

The use of the BFR method has also been evaluated in patients undergoing hemodialysis, particularly given the potential risks of blood vessel injury, which can lead to thrombotic risk and cardiovascular events. In this regard, the study by Corrêa et al.^46^, using intradialytic RT, reported increased D-dimer levels 4 hours after the end of an acute session, highlighting the importance of control and safety mechanisms in the management of this training model. In view of this, the authors recommend assessing variables such as previous cardiovascular events, HD duration, fasting blood glucose levels, age, higher body mass, elevated blood pressure values, and increased C-reactive protein levels as important indicators for avoiding the use of the BFR method^47^. Thus, in light of these findings, even in cases of CKD-3, the use of careful selection criteria for these outcomes is suggested to prevent future complications, along the lines proposed by Corrêa et al.^35^.

In summary, the main contribution of our study is that it is the first to focus specifically on the population with stage 3 CKD, a critical point in the management of the disease, given that many clinical diagnoses, in the absence of renal morphological changes, occur only when the glomerular filtration rate falls below 60 mL/min/1.73 m^2^. Furthermore, examining the use of RT+BFR may shed light on the potential risks associated with its use in clinical practice for managing this population, especially considering that individuals with CKD tend to require RRT at some point during the course of the disease.

Finally, it is worth highlighting the inclusion of this study in a small group of investigations that focus exclusively on RT, since most studies use protocols with aerobic and/or combined exercises in their analyses. Regarding the intervention model, it is worth mentioning the use of a protocol with only 3 exercises per day, which may facilitate adherence to regular physical exercise among this population, as well as the assessment of glomerular function responses in comparison with other intensities, a unique feature of this study.

In terms of limitations, the study did not monitor the participants’ nutrition, although many receive some form of dietary guidance due to their medical conditions from the multidisciplinary team at the institution where they receive clinical care. However, the absence of such monitoring may have led to variations in blood and urinary protein levels, which certainly impacted the fluctuations in eGFR.

Another factor to note is the number of participants, a limitation that may prevent the generalization of the results. However, this does not rule out the possibility that the experiment could serve as a reference or guide for physical education professionals and other healthcare professionals working with populations with similar characteristics.

Although the intensity, the training progression structure, and the design of an exercise protocol were controlled, these factors may have been minimized and/or maximized to provide greater benefits for the participants. Furthermore, the 12-week duration and the lack of long-term monitoring regarding the detraining process and the broader longitudinal effect of the intervention protocol may mask the actual gains and losses observed in this study.

In terms of biochemical tests, the lack of 24-hour proteinuria assessment might have yielded different results regarding urinary protein levels, given that it is the gold standard for rapid screening of renal function decline in the management of conservative treatment. To more accurately assess issues surrounding the safety of the BFR protocol, it would be important to include evaluations of D-dimer, fibrinogen, platelet count, prothrombin time, activated partial thromboplastin time, ferritin, and homeostatic markers.

Finally, the lack of imaging-based assessments of blood vessel injury could elucidate more definitive impacts on safety and changes related to the prevention of significant cardiovascular events associated with the use of the BFR method.

### Practical application

The study revealed that there are no significant changes in glomerular function (creatinine, cystatin C, and eGFR estimates based on these serum markers) in patients with stage 3 CKD when performing MST using the BFR method, especially when compared with other training regimens. Thus, this is a protocol without severe complications for adjuvant treatment in the clinical management of individuals under non-dialytic care. However, additional monitoring of 24-hour proteinuria, nutritional management, and assessment of vascular damage are recommended for better monitoring of glomerular function and cardiovascular events in this population.

## CONCLUSION

After 12 weeks of MST combined with the BFR method in individuals with stage 3 CKD, creatinine and cystatin C levels, as well as glomerular filtration rates, may not show significant differences. However, statistically significant increases in albuminuria levels may occur.

Compared with the other study arms, the BFR group may show statistically significant increases in serum creatinine levels (control and low-load groups) and creatinine-based eGFR (low-load group).

Further studies are expected to better elucidate the impact of MST with BFR in CKD patients.

## Data Availability

The datasets generated and/or analyzed during this study are available upon reasonable request to the corresponding author.
