# Supplementary material for: Effects of muscle strength training with blood flow restriction on glomerular function in people with stage 3 chronic kidney disease
Source: J Bras Nefrol. 2026 Oct 16;48(4):e20260061. doi: 10.1590/2175-8239-JBN-2026-0061en (PMC13680052; doi:10.1590/2175-8239-JBN-2026-0061en)
Supplement: Translation

# Translation: Efeitos do treinamento de força muscular com restrição do fluxo sanguíneo na função glomerular de pessoas com doença renal crônica em estágio 3

**DOI:** 10.1590/2175-8239-JBN-2026-0061pt

**Authors:** Antonio Filipe Pereira Caetano, Janyeliton Alencar de Oliveira, Amaro Wellington da Silva, Cyro Rego Cabral, Michelle Jacintha Oliveira Cavalcante, Maria do Socorro Cirilo-Sousa

**Affiliations:** 1 Universidade Federal de Alagoas, Instituto de Educação Física e Esporte, Maceió, AL, Brasil.; 2 Universidade Federal da Paraíba, João Pessoa, PB, Brasil.; 3 Universidade Federal de Alagoas, Hospital Universitário Professor Alberto Antunes, Maceió, AL, Brasil.; 4 Universidade Federal de Alagoas, Maceió, AL, Brasil.; 5 Universidade Federal de Alagoas, Faculdade de Medicina, Maceió, AL, Brasil.; 6 Universidade Regional do Cariri, Curso de Educação Física, Crato, CE, Brasil.

**Keywords:** Treinamento Resistido, Oclusão Vascular, Insuficiência Renal, Biomarcadores

## Abstract

**Introdução:**

O estágio 3 da doença renal crônica (DRC) representa um momento crítico no tratamento clínico. Todavia, o treinamento de força muscular (TFM) associado ao método de restrição do fluxo sanguíneo (RFS) tem se mostrado benéfico como manejo adjuvante, mas os efeitos renais ainda são incipientes.

**Objetivo:**

Analisar os efeitos do TFM+RFS nos marcadores da função glomerular de pessoas com DRC-3.

**Método:**

Ensaio clínico randomizado, de corte longitudinal, com 40 participantes divididos em 4 grupos: controle (G1); TFM de baixa carga (G2), TFM de alta carga (G3); e TFM de baixa carga associado à RFS (G4). Creatinina sérica, cistatina C, microalbuminúria e estimativa da TFG foram utilizadas como variáveis dependentes. O protocolo de TFM ocorreu durante 12 semanas, 3x/sem, com duração de 30–40 minutos e incluiu 3 exercícios (mono e multiarticulares) por dia, com intensidades progressivas. A análise dos dados (*intention-to-treat*) foi feita com os testes de *Shapiro–Wilk* (normalidade), teste *t* pareado/teste de *Wilcoxon* (comparações intragrupos), ANOVA/*Kruskal–Wallis* (comparação entre grupos), *Bonferroni* (correções *post hoc*) e *d* de Cohen (tamanho do efeito) com valor de *p* ≤ 0,05.

**Resultados:**

Participaram homens (52,5%), com baixos níveis de atividade física (60%), hipertensos (87,5%) e alguns diabéticos (37,5%). O G4 apresentou significância no aumento dos valores de albuminúria (*p* = 0,048), bem como diferenças significativas em comparação ao G1 (creatinina, *p* = 0,032) e G2 (creatinina, *p* = 0,036; e TFG-Creatinina; *p* = 0,032).

**Conclusão:**

O TFM+RFS não demonstrou intercorrências severas nos níveis séricos de creatinina/cistatina C, nas estimativas da TFG e nos níveis de creatinina urinária e microalbuminúria, mas pode aumentar significativamente os valores de albuminúria.

## INTRODUÇÃO

Os rins desempenham inúmeras funções fisiológicas, inclu­indo aspectos hemodinâmicos, hormonais, hematológicos, ósseos, bioquímicos, eletrolíticos e de regulação metabólica^1^. Assim, quando há um comprometimento prolongado, irreversível e persistente de suas funções, instala-se a doença renal crônica (DRC)^2^.

Os níveis de progressão da doença podem ser medidos pela avaliação da função glomerular, determinada pelos valores de excreção de metabólitos (creatinina) e proteínas (albumina) e/ou de células sanguíneas no processo de *clearance*. Durante a filtração, ocorrem a absorção (albumina) e a eliminação de substâncias (resultantes do metabolismo proteico), sendo considerado comprometimento renal quando há diminuição da função dos néfrons^3^.

O insulto renal promove perda irreversível de néfrons, bem como alterações na atividade intrarrenal do sistema renina-angiotensina, contribuindo para o aumento dos níveis pressóricos e do fluxo glomerular. Ao mesmo tempo, devido à hiperfiltração adaptativa, ocorrem manifestações de proteinúria e fibrose renal, culminando em glomeruloesclerose segmentar difusa. Portanto, a partir do *clearance* de substâncias exógenas (inulina, iotalamato-I, EDTA, DTPA-Tc ou ioexol), pode-se identificar com maior precisão a taxa de filtração glomerular (TFG)^4^.

Dessa forma, a TFG pode ser definida como a quantidade de filtração realizada pelos glomérulos por minuto, cujos valores são estimados por diferentes fórmulas^5^. Os estágios 1 a 4 da DRC (TFG >15 mL/min/1,73 m^2^) são considerados para tratamento conservador ou pré-dialítico (manejo medicamentoso e controle do estilo de vida); enquanto no estágio 5 (TFG <15 mL/min/1,73 m^2^), pode-se iniciar a terapia renal substitutiva (TRS) (hemodiálise, diálise peritoneal ou transplante)^6^.

O exercício físico (EF) tem sido recomendado para essa população visando melhorar a função renal^7^, bem como promover alterações morfológicas^8^, aumentar a capacidade funcional^9^, promover ganhos de massa magra^10^ e melhorar a percepção da qualidade de vida^11^. No entanto, o comportamento sedentário e os baixos níveis de atividade física em pessoas com DRC são significativos^12^, por razões ainda pouco investigadas pela comunidade científica, mas que parecem ser semelhantes às observadas na população adulta em geral sem comorbidades associadas^13^.

Dentre as modalidades de EF, o treinamento de força muscular (TFM) tem sido associado ao método de restrição do fluxo sanguíneo (RFS) como forma de proporcionar maiores benefícios, dados os ganhos em massa magra e força muscular promovidos por um número reduzido de exercícios, menor tempo de treino e cargas mais baixas^14^. Para a população com DRC, estudos já reportaram ganhos metabólicos e pressóricos; aumento da força dinâmica; melhora da capacidade funcional; benefícios na vascularização para a confecção de fístula arteriovenosa; regulação da função autonômica; desaceleração do declínio da TFG; alterações na função endotelial, condutância vascular e na complacência venosa; e alterações em marcadores inflamatórios, incluindo IL-18, IL-10, IL-5, TNF-α, vasopressina, F2-isoprostano, NO_2_ e angiotensina-(1–7)^15^.

Todavia, existe uma lacuna de conhecimento no que se refere à prática do EF, aos seus efeitos e à sua relação com a DRC, especialmente em pessoas no estágio 3 (DRC-3) (TFG = 15 a 60 mL/min/1,73 m^2^). Esse estágio é considerado um momento importante no tratamento da doença, tendo em vista que o quadro diagnóstico já se encontra estabelecido, mas apresenta possibilidades de contenção e prevenção da perda da função renal de forma acelerada com mudanças nos hábitos e no estilo de vida^16^. Todos os estudos realizados até o momento concentraram-se nos estágios iniciais (DRC-2) ou finais (DRC-4/5), fases em que a doença pode ainda estar subdiagnosticada e/ou quadros mais graves de alterações podem impedir o uso recorrente da RFS em decorrência de lesão vascular e da futura necessidade de confecção de fístula arteriovenosa para o tratamento hemodialítico.

Além disso, não há estudos que tenham avaliado o efeito do treinamento associado ao método da RFS em comparação com diferentes cargas (alta, baixa e sem carga) para a identificação dos ganhos e/ou perdas dos diferentes métodos de TFM. Isto porque já está consolidado na literatura que o treinamento com uso de intensidades variadas, em populações sem doenças crônicas, impacta diretamente os ganhos de força muscular, massa muscular e os parâmetros bioquímicos^17,18,19^. No entanto, em pessoas com DRC, as condições clínicas, especialmente as restrições proteicas e o tratamento farmacológico, poderiam impactar diretamente esses ganhos. Nesse sentido, mensurar o impacto de um modelo proposto com intensidades variadas pode elucidar caminhos para o desenvolvimento de protocolos específicos para esta população, especialmente em virtude do papel preventivo que a prática de EF pode exercer nos mecanismos de funcionamento e progressão da DRC.

Diante desse contexto, o objetivo deste artigo é analisar os efeitos do TFM associado ao método de RFS sobre a função glomerular de pessoas com DRC estágio 3.

## MÉTODO

Trata-se de um ensaio clínico randomizado com delineamento longitudinal, com grupos paralelos e medidas repetidas. O projeto foi aprovado pelo Comitê de Ética em Pesquisa (CEP) sob o n^o^ 7.159.681/2024 e registrado na Plataforma Registro Brasileiro de Ensaios Clínicos (REBEC), sob a rubrica RBR-7bt6dxb.

### População e amostra

A amostra foi composta por adultos e idosos, de ambos os sexos, com idade entre 35 e 75 anos, com DRC e TFG entre 30 e 59 mL/min/1,73 m^2^, atendidos na Unidade de Sistema Urinário (USU) de um hospital de alta complexidade na cidade de Maceió, Alagoas.

Para verificar a quantidade de sujeitos necessários para o estudo, foi utilizado um cálculo amostral baseado no programa G*Power, adotando um intervalo de confiança de 95% e um erro amostral de 5%, com uma prevalência estimada de pacientes com DRC com TFG entre 30 e 59 mL/ min/1,73 m^2^ atendidos no serviço da USU do HUPAA/UFAL entre os meses de janeiro e julho de 2024, totalizando 197 indivíduos, considerados uma população finita.

Assim, utilizando-se como medida uma potência de 0,80, considerando um nível de significância de 5% e um tamanho de efeito de 0,25 para analisar 4 grupos, realizando 2 medidas em cada um deles, com coeficiente de correlação entre as medidas de 0,50, estimou-se uma amostra de 24 sujeitos. Para evitar perdas amostrais, o número final de participantes do estudo foi ampliado para 40 sujeitos.

A randomização foi realizada por meio de estratificação em blocos, utilizando-se o site http://www.randomizer.org, no qual, por uma sequência oculta, os participantes foram estratificados pelos percentuais dos grupos da pesquisa, sendo distribuídos 25% dos sujeitos em cada grupo. Os participantes foram alocados em 4 grupos: nenhum tipo de TFM, grupo controle (G1); TFM de carga baixa (G2); TFM de carga alta (G3); e TFM de carga baixa associado ao método de RFS (G4).

Os grupos foram pareados por sexo, nível de atividade física, subclassificações do estágio 3 da DRC, diferentes níveis de qualidade de vida e capacidade funcional. A Figura 1 mostra a trajetória dos participantes no processo de seleção, randomização e alocação no estudo.

**Figura 1:** Trajetória dos participantes na seleção, randomização e intervenção.

Abreviaturas – G1: grupo controle; G2: grupo de baixa carga; G3: grupo de alta carga; G4: grupo de restrição de fluxo sanguíneo.

Fontes – Autores, 2025; CONSORT, 2025.

Nesse sentido, foram alocados 40 participantes, sendo 10 sujeitos em cada grupo. Ao término das 12 semanas, 4 indivíduos desistiram do estudo (2 no G2 e 2 no G3) por lesão pré-existente (1), anemia (1) e dificuldade de deslocamento para o local de realização das intervenções (2). A taxa de aderência ao protocolo de treinamento foi de 90%.

A frequência média de todos os participantes (n = 40) foi de 86,5%, sendo 96,5% no G4, 81,7% no G2 e 81,6% no G3.

### Critérios de elegibilidade

O estudo incluiu indivíduos com DRC que residiam próximo à instituição onde o procedimento seria realizado, que assinaram o Termo de Consentimento Livre e Esclarecido (TCLE), que apresentaram liberação médica do acompanhamento nefrológico para fazer parte do estudo e que não apresentavam as seguintes condições clínicas: doenças neurodegenerativas ou infecciosas (HIV/AIDS com carga viral ativa e lúpus eritematoso); insuficiência cardíaca sintomática nas classes II, III e IV, segundo os critérios da *New York Heart Association* (NYHA); sepse nos últimos 12 meses; hepatite B ou C em tratamento; pressão arterial pré-exercício de 160/100 mmHg; disfunção da coagulação ou presença de sinais de tromboflebite; cirurgia nos últimos 3 meses; arritmia grave, angina ou doença cerebrovascular ou cardiovascular; congestão pulmonar ou edema periférico; cirurgia prévia ou acesso vascular nos membros superiores; e participantes que não apresentavam índice tornozelo-braquial (ITB) dentro das faixas recomendadas, como < 0,91 e >1,30 mmHg.

Foram excluídos do estudo aqueles que não compareceram aos testes e à aplicação dos questionários, bem como os pacientes que não obtiveram autorização médica.

### Questionários

Foi aplicado um questionário sociodemográfico e de estilo de vida com perguntas sobre idade, sexo, escolaridade, cor da pele, estado civil, uso de tabaco, álcool e outras drogas e doenças associadas.

Para identificar o nível de atividade física, utilizou-se o IPAQ^20^, com os participantes classificados com base nas recomendações para a prática de atividade física do Guia de Atividade Física para a População Brasileira^21^, a saber: < 150 minutos/semana, considerados baixos níveis de atividade física; ≥ 150 minutos/semana, considerados fisicamente ativos.

### Marcadores da função glomerular

Nas coletas de sangue (soro) e urina para análise da função glomerular (creatinina sérica, cistatina C e microalbuminúria), foram utilizados os métodos cinético/colorimétrico, nefelometria e turbidimetria, respectivamente. Não foram realizados períodos de jejum para as coletas sanguíneas e urinárias.

No momento da coleta, foram obtidos três tubos de sangue e um frasco de urina para a realização de ensaios para determinação de mediadores inflamatórios (dois tubos sem anticoagulante e um com ácido etilenodiaminotetracético/heparina).

A TFG foi calculada com base na fórmula de filtração estimada (eTFG-CKD-EPI), que considera sexo, idade e valores de creatinina sérica (mg/dL), a saber: 142 × min(CrS/ κ,1)^α × max(CrS/κ,1)^-1,200 × 0,9938^Idade × 1,012 (se mulher)^22^, em que CrS representa a concentração de creatinina sérica.

Para a estimativa da filtração com base na cistatina C (eTFG-DRC-Cistatina), foi utilizada a fórmula: 133 × min(CisC/0,8,1)^–0,499 × max(CisC/0,8,1)^–1,328 × 0,996^Idade × 0,932 (se mulher)^23^, em que CisC representa a concentração de cistatina C sérica.

### Avaliações para restrição do fluxo sanguíneo (RFS)

A pressão arterial diastólica e sistólica foi medida após 10 minutos de repouso, com o esfigmomanômetro Premium (ML 177, manguito de náilon 52 × 6 cm e manômetro de 0–300 mmHg), sendo o ponto de corte para a realização da intervenção valores de 160 × 105 mmHg.

A frequência cardíaca e a saturação de oxigênio foram avaliadas após 10 minutos de repouso, utilizando um oxímetro G-Tech Modelo 300C. Os valores de corte para a realização da intervenção foram > 150 bpm e < 70 de SpO_2_.

O ITB foi utilizado como medida preventiva, por ser considerado um importante indicador de risco cardiovascular, com valores adequados na faixa entre 0,91 e 1,30.

Para determinar os valores adequados de RFS, realizou-se a avaliação do pulso auscultatório das artérias braquial (membros superiores) e pediosa (membros inferiores), utilizando um torniquete pneumático Premium, modelo ML177, fixado na região proximal da axila com largura de 52 × 14,5 cm e na região proximal da coxa com largura de 78 × 21 cm, com leitura de 300 mmHg. A ausculta da pressão de insonação foi realizada com o aparelho de Doppler vascular portátil MEDPEJ, de acordo com as recomendações de Gil et al.^24^.

### Teste de 10 repetições máximas

O teste de 10RM seguiu os requisitos para treinamento de força do *American College of Sports Medicine* ^25^, adaptados para populações clínicas^26^. A avaliação foi realizada na 1ª semana, na 6ª (para ajuste da progressão de carga) e após 12 semanas de treinamento.

Inicialmente, foi realizado um aquecimento sem carga para adaptação mecânica ao movimento, aumento da temperatura corporal visando à preparação muscular e à lubrificação das articulações do participante. Em seguida, atribuiu-se uma carga estipulada de forma aleatória para o movimento solicitado, tomada como base inicial do teste. Na sequência, foram executadas séries de 10 repetições, com aumento progressivo de carga para o exercício até a falha concêntrica.

O teste foi interrompido quando o sujeito não conseguia executar corretamente o movimento, sendo considerada como carga máxima a carga mobilizada na última tentativa bem-sucedida. Além disso, foram adotadas as seguintes estratégias para reduzir a margem de erro nos procedimentos de coleta de dados: 1) todos os testes foram realizados na mesma hora do dia, em todas as sessões; 2) instruções padronizadas foram fornecidas antes dos testes, de modo que cada sujeito testado estivesse ciente de toda a rotina de coleta de dados; 3) o sujeito testado foi instruído sobre a técnica adequada da execução do exercício; e 4) todos os participantes receberam encorajamento verbal padronizado durante os testes.

### Protocolo de intervenção

O protocolo de treinamento utilizado está descrito em outro estudo^27^. A intervenção ocorreu ao longo de 12 semanas (36 sessões), com 3 sessões/semana, totalizando 36 sessões. Cada sessão incluiu alongamento (flexão unilateral do cotovelo atrás das costas; flexão unilateral do joelho; flexão do quadril com os dedos tocando o chão; flexão do quadril no espaldar; alongamento da panturrilha na parede), aquec­imento em cicloergômetro (10 min, sem controle de intensidade) e 9 exercícios de força, a saber: supino reto com halteres, extensão do quadril, tríceps na polia alta, remada sentada, mesa flexora, rosca direta, *leg press* 45º, desenvolvimento com halteres e panturrilha no *leg press* 45º.

A periodização do protocolo foi dividida em 4 ciclos, sendo as cargas, séries e repetições ajustadas para equiparação do efeito do treinamento em todos os grupos. Dessa forma, para os G2 e G3, no 1º ciclo (1ª à 3ª semana), foram realizadas 3 séries, de 10 a 12 repetições, com 1 minuto de descanso e cargas de 30% e 60% de 10RM, respectivamente; no 2º ciclo (4ª à 6ª semana), com o mesmo número de séries, repetições e descanso, mas com as cargas ajustadas para 35% e 70% de 10RM; no 3º ciclo (7ª à 9ª semana), foram realizadas 3 séries, de 8 a 10 repetições, com cargas de 40% e 80% de 10RM; e, por fim, no 4º ciclo (10ª à 12ª semana), com 3 séries de 10 a 12 repetições, descanso de 1 minuto e cargas de 40% e 80% de 10RM.

Para o G4, em todos os ciclos, foram realizadas 4 séries de cada exercício, com 15 repetições e carga de 30% de 10RM. Houve um ajuste, na 7ª semana, no valor de RFS, após a realização de uma reavaliação da pressão de insonação. Os exercícios seguiram o tempo marcado por um metrônomo digital, modelo DIGIPOM versão 1.2.0, com emissão de som digital no tema Índigo, com um ritmo de 2 toques no compasso (cadência 2/4), equivalentes a 48 batidas por minuto. A RFS foi feita de maneira condicionante (durante o treinamento), com 50% da pressão arterial diastólica, e realizada de forma contínua. Para a RFS, foram utilizados o aparelho *Hyper Recovery* (material em *nylon*), com dimensões de 68 × 8 cm (membros superiores) e de 108 × 10 cm (membros inferiores), manômetro com coluna de mercúrio 0–300 mmHg, ou aparelho Premium adaptado (material em *nylon*), dimensões 52 × 6 cm (membros superiores) ou 76 × 17 cm (membros inferiores), manômetro com mercúrio de 0–300 mmHg, a depender do tipo de exercício realizado.

A pressão arterial (PA) e a frequência cardíaca (FC) foram medidas antes e depois de cada sessão durante todo o período de intervenção da pesquisa. Durante as sessões de intervenção, a intensidade do exercício foi monitorada por meio da escala de esforço percebido de Borg (PSE-BORG) após o término do número de repetições de cada exercício, ocorrendo o incremento de 0,5 a 1 kg quando havia o reporte de valor abaixo do nível 7 da referida escala.

Por fim, a escala de sensação afetiva ao exercício físico foi aplicada conforme proposto por Hardy e Rejeski^28^ antes e ao final do treinamento.

### Análise estatística

Foi utilizada a análise por intenção de tratar. Todos os participantes randomizados realizaram avaliações pré- e pós-intervenção, não sendo necessária a imputação de dados.

A análise estatística foi conduzida no ambiente R (*R Core Team*, 2024), com o objetivo de investigar comparações intra e intergrupos ao longo do tempo, bem como modelar efeitos fixos e aleatórios por meio de modelos lineares mistos. Foram adotados diferentes testes conforme a estrutura dos dados e as pressuposições de normalidade.

Inicialmente, houve a verificação da normalidade das variáveis (teste de *Shapiro–Wilk)*. Quando a distribuição normal foi confirmada, aplicou-se o teste *t* pareado para comparar as medidas entre os tempos dentro de cada grupo. Nos casos em que a suposição de normalidade não foi atendida, empregou-se o teste de *Wilcoxon* para dados pareados.

A comparação entre os grupos, restrita ao tempo 1 (pós-intervenção), foi realizada utilizando a análise de variância de uma via (ANOVA) para variáveis com distribuição normal. As comparações múltiplas posteriores foram conduzidas com a função TukeyHSD() da base do R. Quando a suposição de normalidade não foi satisfeita, aplicou-se o teste de *Kruskal–Wallis*, e as comparações par a par foram realizadas por meio do teste de *Dunn*, com correção de *Bonferroni*.

As análises mais robustas foram conduzidas por meio de modelos lineares mistos, com intercepto aleatório para os sujeitos, a fim de considerar a estrutura de medidas repetidas no tempo. As significâncias dos coeficientes fixos foram obtidas com auxílio do pacote *lmerTest*, que implementa o método de *Satterthwaite* para o cálculo dos valores de *p*. Foram calculados os tamanhos de efeito, considerando-se elevados os valores acima de 0,8.

Para estimar a magnitude das diferenças entre os tempos dentro de cada grupo, calculou-se o tamanho de efeito por meio do coeficiente *d* de Cohen para medidas pareadas. Os intervalos de confiança de 95% foram obtidos com base na variabilidade das diferenças emparelhadas. Para o tempo 1, os tamanhos de efeito foram estimados com base no coeficiente *d* de Cohen para amostras independentes, utilizando-se como referência o G4 (RFS). Para tanto, foi calculada a diferença entre médias padronizada pelo desvio padrão agrupado, conforme a metodologia tradicional descrita por Cohen. Para a interpretação do tamanho de efeito, utilizou-se a classificação proposta por Rhea: *d* < 0,35 = tamanho de efeito trivial; 0,35 ≤ *d* < 0,8 = baixo tamanho de efeito; 0,8 ≤ *d* < 1,5 = tamanho do efeito moderado; e *d* ≥ 1,5 = grande tamanho de efeito.

Adotou-se um nível de significância de *p* ≤ 0,05 para todas as comparações.

### Acompanhamento clínico

Ao longo das 12 semanas, os participantes do estudo foram supervisionados pelos seus médicos nefrologistas para identificar a manifestação de intercorrências em decorrência do protocolo de treinamento físico proposto, por meio de consultas mensais.

Além disso, a cada 4 semanas, os participantes eram entrevistados para identificar desconfortos, sintomas adversos, lesões físicas estabelecidas e informações sobre as principais dificuldades na adesão ao protocolo. Se houvesse algum tipo de manifestação de complicações vasculares, trombóticas, cardiovasculares ou renais, eles seriam encaminhados ao serviço da USU-HUPAA para maiores verificações.

Se o participante apresentasse algum evento adverso que contraindicasse a continuação na pesquisa, sua participação seria suspensa, e o evento documentado, bem como as informações enviadas ao CEPE. Da mesma forma, o estudo poderia ser suspenso sempre que o CEPE entendesse ser necessária a suspensão ou o encerramento da pesquisa, conforme preconizam os princípios éticos das Resoluções nº 466/2012 e nº 510/2016 do Conselho Nacional de Saúde.

## RESULTADOS

Quarenta indivíduos participaram do estudo (52,5% homens, idade de 58 ± 8,9 anos). O perfil sociodemográfico e de saúde está detalhado na Tabela 1. A maioria era hipertensa (87,5%), 37,5% apresentavam diabetes *mellitus* e 60% foram classificados como tendo baixos níveis de atividade física.

**Tabela 1:** Características sociodemográficas e estilo de vida de pacientes com DRC-3.

Variáveis	G1 N = 10 (%)	G2 N = 10 (%)	G3 N = 10 (%)	G4 N = 10 (%)	Geral N = 40 (%)
Sexo					
Homens	5 (50,0)	5 (50,0)	6 (60,0)	5 (50,0)	21 (52,5)
Mulheres	5 (50,0)	5 (50,0)	4 (40,0)	5 (50,0)	19 (47,5)
Cor da pele					
Parda	5 (50,0)	5 (50,0)	1 (10,0)	7 (70,0)	18 (45,0)
Preta	3 (30,0)	2 (20,0)	4 (40,0)	1 (10,0)	10 (25,0)
Branca	2 (20,0)	2 (20,0)	4 (40,0)	1 (10,0)	9 (22,5)
Indígena	–	1 (10,0)	1 (40,0)	1 (10,0)	3 (7,5)
Estado civil					
Solteiro	5 (50,0)	2 (20,0)	2 (20,0)	2 (20,0)	11 (27,5)
Casado	4 (40,0)	7 (70,0)	7 (70,0)	6 (60,0)	24 (60,0)
Viúvo	1 (10,0)	–	–	1 (10,0)	2 (6,25)
Divorciado	–	1 (10,0)	1 (10,0)	1 (10,0)	3 (6,25)
Exerce ocupação profissional					
Sim	3 (30,0)	2 (20,0)	3 (30,0)	6 (60,0)	14 (35,0)
Nível de escolaridade					
Fundamental incompleto	3 (30,0)	7 (70,0)	5 (50,0)	2 (20,0)	17 (42,5)
Fundamental completo	2 (20,0)	1 (10,0)	–	3 (20,0)	6 (15,0)
Ensino Médio	2 (20,0)	–	5 (50,0)	3 (30,0)	10 (25,0)
Analfabeto	1 (10,0)	1 (10,0)	–	–	2 (15,0)
Ensino superior	2 (20,0)	1 (10,0)	–	2 (20,0)	5 (2,5)
Diabetes					
Sim	4 (40,0)	4 (40,0)	5 (50,0)	2 (20,0)	15 (37,5)
Hipertensão arterial					
Sim	8 (80,0)	8 (80,0)	9 (90,0)	10 (100,0)	35 (87,5)
Outras doenças					
Sim	7 (80,0)	4 (40,0)	3 (30,0)	4 (40,0)	18 (45,0)
Tabagismo					
Sim	1 (10,0)	4 (40,0)	3 (30,0)	2(20,0)	10 (25,0)
Não	9 (90,0)	5 (50,0)	6 (60,0)	8 (80,0)	28 (70,0)
Ex-fumante	–	1 (10,0)	1 (10,0)	–	2 (5,0)
Consumo de álcool					
Sim	–	3 (30,0)	3 (30,0)	3 (30,0)	9 (22,5)
Não	9 (90,0)	4 (40,0)	5 (50,0)	3 (30,0)	21 (52,5)
Ex-consumidor	1 (10,0)	3 (30,0)	2 (20,0)	4 (40,0)	10 (25,0)
Estágio DRC					
3a	4 (40,0)	3 (30,0)	5 (50,0)	1 (10,0)	13 (32,5)
3b	6 (60,0)	7 (70,0)	5 (50,0)	9 (90,0)	27 (67,5)
Nível de atividade física					
Sedentário	5 (50,0)	5 (50,0)	1 (10,0)	2 (20,0)	13 (32,5)
Insuficientemente ativo	2 (10,0)	3 (30,0)	4 (40,0)	2 (20,0)	11 (27,5)
Ativo	–	–	3 (30,0)	3 (20,0)	6 (15,0)
Muito ativo	3 (40,0)	2 (20,0)	2 (20,0)	3 (30,0)	10 (25,0)

Abreviaturas – G1: grupo controle; G2: grupo de baixa carga; G3: grupo de alta carga; G4: grupo de restrição de fluxo sanguíneo; N: número; %: porcentagem; DRC: doença renal crônica.

Fonte – Autores, 2024.

A comparação das variáveis intragrupo após a intervenção pode ser observada na Tabela 2, e um resumo dos resultados dessas comparações pode ser visto na Figura 2.

**Tabela 2:** Comparação das médias/medianas intragrupos dos marcadores de função glomerular após 12 semanas do protocolo de intervenção.

Variáveis	G1					G2					G3					G4				
	T0	T1	∆	1-β	*p*-valor	T0	T1	∆	1-β	*p*-valor	T0	T1	∆	1-β	*p*-valor	T0	T1	∆	1-β	*p*-valor
Cr (mg/dL)	1,34 ± 0,26	1,39 ± 0,26	+0,05	0,14	0,010*	1,30 ± 0,41	1,29 ± 0,26	−0,01	0,05	0,322	1,57 ± 0,52	1,71 ± 0,64	+0,14	0,08	0,031*	1,90 ± 0,7	2,06 ± 0,92	+0,16	0,11	0,066
Cist C (mg/L)	1,63 ± 0,43	1,51 ± 0,34	−0,12	0,10	0,071	1,57 ± 0,46	1,47 ± 0,40	−0,10	0,05	0,556	1,65 ± 0,57	1,67 ± 0,47	+0,02	0,06	0,845	1,8^‡^ [1,5–1,9]	1,9 ± 0,6	+0,10	0,05	0,049
MAU_Cr (mg/dL)	69,0 ± 30,4	106,1 ± 40,5	+37,1	0,31	0,005*	73,0‡ [44,0–181,0]	89,0 ± 41,3	+16,0	0,10	0,463	107,4 ± 78,3	111,3 ± 51,9	+3,9	0,05	0,808	115,7 ± 45,9	92,4 ± 42,8	−23,3	0,07	0,190
MAU_Alb (mg/L)	127,8 ± 84,9	144,5^‡^ [9,9–593,8]	+16,7	0,07	0,492	34,6‡ [5,9–1238,0]	178,7‡ [7,0–1329]	+144,1	0,12	0,598	40,7‡ [6,5–1866]	24,7‡ [7,0–1365]	−16,0	0,09	0,315	101,9‡ [9,0–709,7]	174,0 ± 171,0	+72,1	0,06	0,048*
MAU_RAC (mg/g creatinina)	218,5^‡^ [16,0–609,0]	131,5 ± 103,8	−87,0	0,10	0,048*	44,0‡ [8,0–2211]	42,5‡ [8,0–1642]	−1,5	0,11	0,050*	56,0‡ [7,0–1048]	23,0‡ [6,0–1219]	−33,0	0,11	1,000	103,8 ± 53,7	127,0‡ [10–173]	+22,2	0,05	0,845
TFG-Cr (mL/min/ 1,73 m*2*)	46,4 ± 9,7	44,5 ± 9,8	−1,9	0,78	0,010*	57,0‡ [51,0–60,0]	52,1 ± 6,2	−4,9	0,05	0,495	45,7 ± 13,6	43,4 ± 15,4	−2,3	0,40	0,120	39,6 ± 13,2	37,4 ± 14,9	−2,2	0,55	0,066
TFG-C (mL/min/ 1,73 m2)	47,4 ± 19,3	52,9 ± 16,1	+5,5	0,06	0,020*	48,1 ± 14,9	51,8‡ [48,6–60,9]	+3,7	0,08	0,089	49,7‡ [27,6–59,6]	41,0 ± 15,4	−8,7	0,25	0,179	38,1 ± 16,7	33,6‡ [22,8–38,3]	−4,5	0,05	0,781

Abreviaturas – G1: grupo controle; G2: grupo de baixa intensidade; G3: grupo de alta intensidade; G4: grupo de restrição de fluxo sanguíneo; T0: pré-intervenção; T1: pós-intervenção; Cr: creatinina sérica; Cist C: cistatina C; MAU-Cr: microalbuminúria–creatinina; MAU_Alb: microalbuminúria–albumina; MAU_RAC: microalbuminúria–razão albumina/creatinina; TFG-Cr: estimativa da taxa de filtração glomerular (creatinina); TFG-C: estimativa da taxa de filtração glomerular (cistatina C).

Notas – (‡): mediana [intervalo interquartil];

(*): *p*-valor < 0,05 intragrupos; 1-β: efeito *post hoc*; ∆: diferença entre os tempos.

Fonte – Autores, 2025.

**Figura 2:** *Boxplot* dos resultados da função glomerular intragrupos.

O G1 apresentou significância estatística nas variáveis creatinina sérica (*p* = 0,010; *d* = 1,13); relação microalbuminúria/creatinina (*p* = 0,005), relação creatinina/albumina (*p* = 0,048; *d* = 1,15) e estimativa da TFG-Creatinina (*p* = 0,048; *d* = -0,84) e da TFG-Cistatina C (*p* = 0,20; *d* = 0,89). Em todos os casos, o tamanho de efeito pode ser considerado grande. Porém, somente nas variáveis relação creatinina/albumina e estimativa da TFG-Cistatina C as alterações foram benéficas.

No grupo baixa carga (G2), apenas o resultado para microalbuminúria/creatinina apresentou melhora nas médias/medianas com tendência à significância (*p* = 0,050; *d* = 0,80), com tamanho de efeito grande.

Para o G3 (alta carga), observou-se um aumento da expressão de creatinina (*p* = 0,031; *d* = 0,80), com tamanho de efeito grande.

Para o grupo com RFS (G4), houve aumento nos valores médios de albumina urinária (*p* = 0,048; *d* = -0,4), mas com tamanho de efeito moderado.

Na comparação entre os grupos, tendo como referência o G4 (Tabela 3), os dados demonstraram diferença significativa na variável creatinina sérica entre G1 (*p* = 0,032; *d* = 0,62) e G2 (*p* = 0,036; *d* = 0,77), com tamanhos de efeito moderados.

**Tabela 3:** Comparação da função glomerular entre os grupos após 12 semanas do protocolo de intervenção.

Variáveis	G1 × G4				G2 × G4				G3 × G4			
	Coef	*p*-valor	1-β	IC95%	Coef	*p*-valor	1-β	IC95%	Coef	*p*-valor	1-β	IC95%
Cr (mg/dL)	−0,479	0,032*	0,05	−0,917; −0,042	−0,479	0,036*	0,20	−0,928; −0,031	−0,170	0,457	0,05	−0,617; 0,277
Cist C (mg/L)	−0,277	0,216	0,13	0,715; 0,162	−0,265	0,250	0,06	−0,716; 0,186	−0,121	0,597	0,05	−0,570; 0,328
MAU_Cr (mg/dL)	−8,254	0,660	0,07	−45,066; 28,558	−13,818	0,474	0,05	−51,655; 24,018	7,531	0,695	0,14	30,150; 45,212
MAU_Alb (mg/L)	−32,053	0,829	0,23	−323,194; 259,088	130,696	0,392	0,05	−168,418; 429,810	189,818	0,212	0,24	312,202; 1509,892
MAU_RAC (mg/g creatinina)	−98,374	0,563	0,05	−431,312; 234,565	140,96	0,419	0,07	−201,100; 483,023	134,83	0,438	0,23	−205,84; 475,512
TFG-Cr (mL/min/1,73 m^2^)	6,213	0,244	0,28	−4,28; 16,674	11,284	0,039*	0,96	0,547; 22,022	3,425	0,530	0,16	−5,04; 16,17
TFG-C (mL/min/1,73 m^2^)	10,482	0,190	0,11	−5,195; 26,160	11,824	0,150	0,40	4,28; 27,933	2,892	0,724	0,05	−13,151; 18,936

Abreviaturas – G1: grupo controle; G2: grupo de baixa intensidade; G3: grupo de alta intensidade; G4: grupo de restrição de fluxo sanguíneo; T0: pré-intervenção; T1: pós-intervenção; Cr: creatinina sérica; Cist C: cistatina C; MAU_Cr: microalbuminúria–creatinina; MAU_Alb: microalbuminúria–albumina; MAU_RAC: microalbuminúria–razão albumina/creatinina; TFG-Cr: estimativa da taxa de filtração glomerular (creatinina); TFG-C: estimativa da taxa de filtração glomerular (cistatina C).

Notas – (*): *p*-valor < 0,05 entre os grupos; 1-β: efeito *post hoc*; ∆: diferença entre os tempos.

Fonte – Autores, 2025.

Na estimativa da TFG baseada em creatinina, também houve diferença estatística em relação ao G2 (*p* = 0,039; *d* = 1,29), com tamanho de efeito grande.

A taxa média de comparecimento às sessões de treinamento foi de 86,5%, sendo maior no G4 (96,5%).

Em relação à sensação afetiva ao exercício, apenas o G3 (carga alta) apresentou melhora significativa na escala (*p* = 0,031). G2 e G4 apresentaram melhoras nas médias, mas sem significância estatística.

Em relação aos efeitos adversos, foram relatados pelos participantes casos leves e transitórios de dor no pescoço (primeiras semanas), queimação nos braços, fadiga nos membros e coceira na pele. Não foram relatados eventos adversos graves como complicações cardiovasculares, eventos trombóticos ou complicações renais clinicamente relevantes durante o período de intervenção.

## DISCUSSÃO

Após 12 semanas de TFM, verificou-se que os participantes que utilizaram o protocolo de RFS não apresentaram alterações significativas nas concentrações séricas de creatinina e cistatina C, bem como nas estimativas da TFG (TFGe) com esses marcadores.

Isso pode ter ocorrido em virtude da necessidade de geração rápida de energia muscular (ATP), de modo que a creatina fosfato (fosfocreatina) é degradada em creatinina na corrente sanguínea. Assim, devido à ação muscular constante durante as tarefas, a excreção de creatinina é permanente no organismo^1,29^. No entanto, quando ocorrem problemas no processo de filtração renal, a incidência de danos à membrana basal glomerular torna-se latente^2^. O TFM tende a aumentar o uso de moléculas de ATP (trifosfato de adenosina), seja por fosforilação oxidativa nas mitocôndrias, glicólise anaeróbica ou fosforilação em nível de substrato no citoplasma e nas mitocôndrias^30^. Esse sistema é desencadeado agudamente pelo próprio sistema nervoso central, que promove a ativação muscular com o recrutamento de unidades motoras do tipo I (lentas-vermelhas) e, subsequentemente, fibras do tipo II (rápidas-brancas)^31^.

No entanto, com o uso do método RFS, esse sistema é potencializado pela hipóxia, que atua na aceleração do recrutamento de fibras rápidas, causando um efeito em cascata, com aumento das alterações metabólicas e estímulo à liberação de hormônios como testosterona, GH e cortisol^32^. Os impactos fisiológicos descritos, associados aos estresses mecânicos que proliferam lesões de micro cisalhamento na fibra, podem possibilitar ganhos em hipertrofia e força muscular^33^. Isso talvez explique o aumento, ainda que sem significância estatística, da creatinina sérica nos grupos G3 e G4 no presente estudo, considerando que fatores como intensidade, número de repetições e tempo de descanso do exercício influenciam diretamente o metabolismo energético. Além disso, em comparação com os grupos G1 e G2, o grupo G4 apresentou diferença estatisticamente significativa nas concentrações séricas de creatinina, ainda que esses dois grupos tenham apresentado diferenças no comportamento desse metabólito, com aumento (G1) e diminuição (G2) dos valores médios após o período de intervenção.

Essa hipótese pode ser sustentada pelos próprios resultados na TFGe baseada em creatinina, em que o G1 foi o único braço do estudo que manifestou diferenças (diminuição nos valores médios) entre os momentos, embora não tenha apresentado diferença estatística em relação ao G4. Em contrapartida, o G2 apresentou uma diminuição nos valores médios da estimativa da TFGe-Creatinina e uma diferença significativa em relação ao G4. Assim, mesmo sabendo que a equação considera o sexo e a idade, os resultados podem indicar que o TFM+RFS pode contribuir para conter o aumento expressivo da creatinina sérica e a diminuição da TFGe-Creatinina em pessoas com DRC-3.

O uso do método RFS em pessoas com DRC-3 ainda é pouco avaliado na literatura. No caso de indivíduos no estágio 2, Corrêa et al.^34^ analisaram 229 pacientes com um protocolo de TFM de 6 meses e observaram os mesmos achados de manutenção dos níveis de creatinina com o uso da RFS e aumento em outras intensidades. Esse mesmo autor, em outra análise envolvendo 105 indivíduos com DRC-2, propondo um modelo de TFM com e sem RFS, percebeu uma atenuação da TFGe e dos níveis urêmicos, bem como uma melhora no perfil inflamatório e no eixo Klotho-FGF23 — um marcador destacado para o controle dos níveis de fósforo e a mitigação de danos cardiovasculares e ósseos — naqueles expostos ao exercício em comparação com o grupo controle, o qual, em contrapartida, apresentou progressão para DRC-3 ao término da investigação^35^. Tais resultados, somados aos nossos achados sobre os efeitos na TFGe, podem indicar o TFM como um importante aliado na contenção do avanço mais acelerado da DRC em termos de filtração sanguínea, sendo a intensidade um elemento a ser observado, uma vez que o treinamento com baixas intensidades apresentou significância na diminuição da TFGe.

Ainda nessa perspectiva, Deus et al.^36^, ao analisar a função autonômica cardíaca em 105 pessoas com DRC-2, também detectaram melhorias na interrupção do declínio da função renal, a partir de possíveis respostas positivas no equilíbrio redox, nas enzimas inflamatórias paraoxonase 1 (PON1) e mieloperoxidase (MPO) e na modulação autonômica cardiovascular, com o uso de TFM com ou sem RFS. Em outro estudo envolvendo 90 pacientes adultos e idosos, Corrêa et al.^37^ identificaram alterações na expressão de angiotensina-1, vasopressina e óxido nítrico nos grupos com RFS, situação que poderia afetar as elevações nos níveis de creatinina, dada a resposta metabólica natural do organismo. Em consonância, estes estudos, que avaliaram as interações cardiorrenais com maior profundidade, permitem-nos explicar as possíveis oscilações nos níveis séricos de creatinina e da TFGe na DRC-3, reforçando o papel do TFM como agente estressor e possível modulador dos mecanismos hemodinâmicos, do sistema nervoso simpático, da disfunção endotelial, da expressão de NO_2_, da inflamação sistêmica, do sistema renina-angiotensina e dos desequilíbrios eletrolíticos, independentemente do uso da RFS.

Sem o uso do TFM+RFS, achados anteriores já relataram ganhos na TFGe, nos níveis de creatinina sérica e nos níveis de proteinúria em protocolos que associaram TFM ao treinamento aeróbico de intensidades variadas e ao uso de implementos, além de analisarem diferentes estágios da DRC, do estágio 2 ao estágio 4^38^. O não isolamento do estágio 3 da DRC provavelmente impede a identificação de respostas mais precisas sobre os efeitos do TFM nessa população, bem como a associação com altos níveis de treinamento aeróbico pode mascarar resultados potenciais em termos de TFGe.

No entanto, devido à influência exercida pela massa muscular, ingestão de proteínas, estado hídrico e práticas habituais de exercício físico nos níveis de creatinina sérica, a cistatina C torna-se um biomarcador complementar essencial para a avaliação da função glomerular na DRC. A cistatina C está presente em várias células do organismo, é responsável pelos processos de degradação de proteínas, funciona como um regulador das atividades de protease, contribui para a remodelação das matrizes extracelulares, auxilia na regulação dos distúrbios inflamatórios e na proteção imunológica e atua na degradação de proteínas intracelulares^39^.

Assim, sob condições naturais, a cistatina C não é excretada de forma significativa no processo de filtração glomerular, dada sua reabsorção e degradação quase completas nos túbulos renais^40^. No contexto da prática do EF, essa proteína, durante atividades mais intensas, pode ter seus níveis séricos aumentados, especialmente devido a intercorrências situacionais como lesão muscular, perda de líquidos e elevação de marcadores inflamatórios, como IL-6 e TNF-α. Porém, cronicamente, o EF tende a minimizar o aumento da expressão de cistatina C, ajudando a estabilizar os níveis inflamatórios e proporcionando uma melhor TFGe^41^.

Em nossos achados, em nenhum grupo houve diferença significativa nos valores médios na expressão de cistatina C após 12 semanas de exposição ao protocolo proposto.

Esse é um achado interessante, tendo em vista que, independentemente da carga do TFM, o estresse muscular e a pressão exercida sobre os vasos sanguíneos podem não ser capazes de promover mudanças significativas nessa proteína. Todavia, cabe mencionar as características fisiológicas de pessoas com DRC, que tendem a apresentar níveis elevados de pressão arterial, o que, em alguns casos, pode levar ao comprometimento da função óssea e à alta retenção de líquidos, especialmente no estágio 3. Esse conjunto de condições clínicas talvez possa influenciar na adaptação mais lenta do organismo às respostas adaptativas promovidas pelo TFM na regulação da expressão de cistatina C sérica e da filtração glomerular. Côrrea et al.^34^ também identificaram um aumento nos níveis de cistatina C em seu protocolo para pessoas com DRC-2, o que pode, de fato, indicar uma tendência de elevação dessa proteína no treinamento por curtos períodos (3 a 6 meses).

No entanto, a não exposição ao TFM pode ter contribuído para as mudanças positivas e significativas na estimativa da TFGe-Cistatina C no G1, no qual ocorreu aumento da TFGe. Por outro lado, observaram-se oscilações nos valores médios, sem significância estatística, nos demais grupos do estudo.

Sabendo que a TFGe é um marcador para determinar o avanço dos estágios da DRC, é necessário salientar que o único grupo que teria mudado o estrato de classificação da TFGe após o período de intervenção foi o G3, passando do estágio 3a para o estágio 3b, tanto usando creatinina quanto cistatina C para o cálculo. O G1 mudaria apenas de estrato (3a para 3b) utilizando a TFGe-Creatinina.

Para verificar essa perda acelerada da função renal, o método da microalbuminúria por excreção urinária é um recurso importante. O uso da albumina como marcador detecta agudamente danos ao glomérulo, especialmente devido a doenças associadas, como diabetes mellitus (DM) e hipertensão arterial sistêmica (HAS), mas também está presente em outras comorbidades como HIV, lúpus, hepatite e glomerulonefrite. Por estar presente no plasma sanguíneo, a albumina auxilia no equilíbrio da pressão oncótica, no transporte de substâncias solúveis em água (tiroxina, cortisol, ácidos graxos livres, bilirrubina, cálcio, magnésio e anti-inflamatórios), na reposição de aminoácidos durante o suporte metabólico, na regulação dos níveis de pH do sangue e na minimização do estresse oxidativo, neutralizando a ação dos radicais livres^41^. Em nossos achados, na relação albumina/creatinina urinária, o G1 e o G2 apresentaram melhoras significativas, mas, independentemente do grupo no qual os resultados dessa microalbuminúria foram identificados, os valores médios estavam fora dos padrões normais (< 30 mg/L).

A ação do TFM sobre a microalbuminúria está relacionada, acima de tudo, às condições clínicas de seus praticantes. Assim, treinamentos com intensidades mais altas promovem respostas mais expressivas nos valores de microalbuminúria, impactando o fluxo sanguíneo renal, aumentando os parâmetros hemodinâmicos, elevando o estresse oxidativo e alterando as respostas inflamatórias. No entanto, com intensidades mais controladas e quando realizado cronicamente, o exercício pode ajudar a melhorar a função endotelial e, consequentemente, a regular a pressão arterial e a reduzir os níveis de microalbuminúria.

Nesse sentido, podemos considerar a hipótese de que o uso do método de RFS, devido à oclusão vascular e ao consequente acúmulo de metabólitos, pode ter potencializado as alterações negativas e significativas dos níveis de albuminúria. Desta feita, a vasoconstrição pode ter diminuído o suprimento de oxigênio e nutrientes, aumentando a resistência vascular periférica e os valores da PA, que, ao desencadearem a ativação do sistema nervoso simpático, contribuíram para alterações hormonais (endotelina-1, angiotensina II, vasopressina e catecolaminas)^30,33^.

Hamada et al.^42^, combinando TFM e treinamento aeróbico em pessoas em tratamento conservador (DRC-2–DRC-4), notaram uma redução estatisticamente significativa nos níveis de proteinúria nesses pacientes, resultados que não foram identificados por Hiraki et al.^43^ e Zhang et al.^44^, que, embora tenham observado um aumento nos valores de proteinúria no grupo experimental, não demonstraram significância estatística. Em relação aos estudos que utilizam o método RFS, não há inclusão desse marcador nas análises existentes.

Nos resultados de nossa investigação, não foi feita uma associação entre a função glomerular e as condições glicêmicas dos participantes. Todavia, Deus et al.^45^, ao se debruçarem sobre pessoas com DRC-2, com um modelo de TFM com ou sem RFS durante 6 meses, perceberam que, ao mesmo tempo em que houve uma diminuição da TFGe, os participantes também apresentaram melhoras nos níveis de homeostase glicêmica, nos mediadores hormonais de captação de glicose e no perfil imunometabólico. Tais achados sinalizam a associação de mecanismos metabólicos e renais na contenção da progressão da DRC e reforçam o TFM como um modulador essencial para ganhos adicionais no tratamento conservador, sobretudo no controle das doenças de base, como DM e HAS.

O uso do método de RFS também tem sido avaliado em pacientes em tratamento hemodialítico, especialmente ao se perceber o potencial risco de comprometimento dos vasos sanguíneos, que pode levar ao risco trombótico e a eventos cardiovasculares. Nesse sentido, a investigação proposta por Corrêa et al.^46^, com TFM intradialítico, reportou aumento dos níveis de dímero D 4 horas após o término de uma sessão aguda, lançando luz sobre a importância de mecanismos de controle e segurança no manejo deste modelo de treinamento. Diante disso, os autores recomendam a averiguação de variáveis como eventos cardiovasculares prévios, tempo de HD, níveis de glicemia de jejum, idade, maior massa corporal, valores elevados de pressão arterial e proteína C-reativa aumentada como importantes sinalizadores para a não utilização do método de RFS^47^. Assim, diante do exposto, mesmo em casos de DRC-3, sugere-se o uso de modelos de seleção criteriosa para estes desfechos, a fim de evitar intercorrências futuras, nos mesmos moldes propostos por Corrêa et al.^35^.

Em síntese, a principal contribuição de nosso estudo é ser o primeiro a focar especificamente na população com DRC-3, um momento importante no tratamento da doença, considerando que muitos diagnósticos clínicos, sem a presença de alterações morfológicas renais, ocorrem apenas com filtração abaixo de 60 mL/min/1,73 m^2^. Além disso, verificar o uso do TFM+RFS pode elucidar riscos potenciais de sua utilização na prática profissional para o manejo dessa população, especialmente considerando que indivíduos com DRC tendem a fazer uso da TRS em algum momento do tratamento da doença.

Por fim, vale destacar a inclusão deste estudo em um pequeno grupo de investigações que focam exclusivamente no TFM, visto que a maioria dos trabalhos utiliza protocolos com exercícios aeróbicos e/ou combinados em suas análises. No que tange ao modelo de intervenção, vale mencionar o uso de um protocolo com apenas 3 exercícios por dia, o que pode facilitar a adesão à prática regular de exercício físico por essa população, bem como a verificação das respostas da função glomerular em comparação com outras intensidades, um marco original desta investigação.

Em termos de limitações, o estudo não realizou controle nutricional dos participantes, embora muitos tenham algum tipo de acompanhamento dietético, devido às condições da doença, pela própria equipe multidisciplinar da instituição onde recebem acompanhamento clínico. No entanto, a ausência desse tipo de controle pode ter levado a diferentes expressões de proteínas sanguíneas e urinárias, impactando, certamente, as oscilações da TFGe.

Outro elemento a ser relatado é o número de participantes, condição que pode impedir a extrapolação desta análise de forma mais geral. Entretanto, isso não exclui a possibilidade de o experimento ser utilizado como referência ou guia para profissionais de Educação Física e outros profissionais de saúde que trabalham com populações com as mesmas características.

Embora tenha havido controle de intensidade, da estrutura de progressão do treinamento e da elaboração de um protocolo de exercícios, esses aspectos podem ter sido minimizados e/ou maximizados para maiores benefícios aos participantes. Além disso, o período de 12 semanas e a falta de verificação a longo prazo, do processo de destreinamento e do efeito longitudinal mais alargado do protocolo de intervenção podem mascarar as perdas/ganhos reais dessa investigação.

Em termos de exames bioquímicos, o não uso da análise de proteinúria de 24 horas poderia ter revelado resultados diferentes nos níveis de proteína urinária, considerando que esse exame é o padrão ouro para a triagem acelerada da perda de função renal no manejo do tratamento conservador. Para verificar de forma mais precisa as questões em torno da segurança do protocolo de RFS, seria importante incluir avaliações de dímero D, fibrinogênio, contagem de plaquetas, tempo de protrombina, tempo de tromboplastina parcial ativada, ferritina e marcadores homeostáticos.

Por fim, o não uso de avaliações de danos nos vasos sanguíneos através de imagens, que poderiam elucidar impactos mais assertivos sobre a segurança e as alterações relacionadas à contenção de eventos cardiovasculares significativos associados ao uso do método de RFS.

### Aplicação prática

O estudo revelou que não ocorrem alterações significativas na função glomerular (creatinina, cistatina C e estimativas da TFG com base nesses marcadores séricos) de pessoas com DRC estágio 3 ao realizar TFM associado ao método RFS, especialmente em comparação com programas que utilizam outras cargas. Dessa forma, este é um protocolo sem intercorrências severas para o tratamento adjuvante no manejo clínico de indivíduos sob acompanhamento não dialítico. No entanto, recomenda-se o monitoramento adicional da proteinúria de 24 horas, o controle nutricional e a avaliação de danos vasculares para melhor acompanhamento do manejo da função glomerular e dos eventos cardiovasculares nessa população.

## CONCLUSÃO

Após 12 semanas de TFM associado ao método RFS em indivíduos com DRC estágio 3, os níveis de creatinina, cistatina C e as taxas de filtração glomerular podem não apresentar diferenças significativas. No entanto, podem ocorrer elevações nos níveis de albuminúria com significância estatística.

Comparado aos outros braços do estudo, o grupo RFS pode apresentar aumentos nas expressões de creatinina sérica (controle e carga baixa) e na TFGe baseada em creatinina (grupo carga baixa) com significância estatística.

Espera-se que estudos futuros possam elucidar melhor o impacto do TFM com RFS em pacientes com DRC.

## Data Availability

Os conjuntos de dados gerados e/ou analisados durante o presente estudo estão disponíveis mediante solicitação justificada ao autor correspondente.
